# Long-read transcriptome sequencing reveals abundant promoter diversity in distinct molecular subtypes of gastric cancer

**DOI:** 10.1186/s13059-021-02261-x

**Published:** 2021-01-22

**Authors:** Kie Kyon Huang, Jiawen Huang, Jeanie Kar Leng Wu, Minghui Lee, Su Ting Tay, Vikrant Kumar, Kalpana Ramnarayanan, Nisha Padmanabhan, Chang Xu, Angie Lay Keng Tan, Charlene Chan, Dennis Kappei, Jonathan Göke, Patrick Tan

**Affiliations:** 1 Programme in Cancer and Stem Cell Biology, Duke-NUS Medical School, 8 College Road, Singapore, 169857 Singapore; 2 Cancer Science Institute of Singapore, National University of Singapore, Singapore, 117599 Singapore; 3 Department of Biochemistry, Yong Loo Lin School of Medicine, National University of Singapore, Singapore, 117596 Singapore; 4 Genome Institute of Singapore, Singapore, 138672 Singapore; 5 SingHealth/Duke-NUS Institute of Precision Medicine, National Heart Centre Singapore, Singapore, 169609 Singapore

**Keywords:** Gastric cancer, Alternative splicing, Alternative promoter, Iso-seq

## Abstract

**Background:**

Deregulated gene expression is a hallmark of cancer; however, most studies to date have analyzed short-read RNA sequencing data with inherent limitations. Here, we combine PacBio long-read isoform sequencing (Iso-Seq) and Illumina paired-end short-read RNA sequencing to comprehensively survey the transcriptome of gastric cancer (GC), a leading cause of global cancer mortality.

**Results:**

We performed full-length transcriptome analysis across 10 GC cell lines covering four major GC molecular subtypes (chromosomal unstable, Epstein-Barr positive, genome stable and microsatellite unstable). We identify 60,239 non-redundant full-length transcripts, of which > 66% are novel compared to current transcriptome databases. Novel isoforms are more likely to be cell line and subtype specific, expressed at lower levels with larger number of exons, with longer isoform/coding sequence lengths. Most novel isoforms utilize an alternate first exon, and compared to other alternative splicing categories, are expressed at higher levels and exhibit higher variability. Collectively, we observe alternate promoter usage in 25% of detected genes, with the majority (84.2%) of known/novel promoter pairs exhibiting potential changes in their coding sequences. Mapping these alternate promoters to TCGA GC samples, we identify several cancer-associated isoforms, including novel variants of oncogenes. Tumor-specific transcript isoforms tend to alter protein coding sequences to a larger extent than other isoforms. Analysis of outcome data suggests that novel isoforms may impart additional prognostic information.

**Conclusions:**

Our results provide a rich resource of full-length transcriptome data for deeper studies of GC and other gastrointestinal malignancies.

**Supplementary Information:**

The online version contains supplementary material available at 10.1186/s13059-021-02261-x.

## Background

Gastric cancer (GC) is the fifth leading type of cancer and third leading cause of cancer death, with more than 1.03 million new cases and 780,000 deaths reported annually [1]. Traditional classification systems based on tumor morphology such as the Lauren (diffuse and intestinal) [2] and the World Health Organization systems (papillary, tubular, mucinous and poorly cohesive) [3] have limited clinical utility in guiding the treatment of GC patients. This has led to attempts to apply high-throughput molecular methods to classify GC into molecular subtypes that can convey more detailed information compared to histological methods.

Among different molecular platforms, conventional short-read RNA sequencing has been widely used to identify transcripts and gene expression changes in GC [4–6]. While this method has been effective in quantifying transcript abundance, short reads (usually 100 to 250 base pair) rarely span full-length transcripts, which can often be several kilobases long, making it difficult to directly infer full-length transcript structure. These limitations are particularly pronounced in complex human transcriptomes such as GC, which may express many distinct but very similar isoforms resulting from different alternative promoters, exons, and 3′ untranslated regions (UTRs) [7, 8]. As a result, the full-length transcriptome of GC has remained under-explored, despite the potential importance of this information to understand the biological roles of alternative isoforms.

Recently, single-molecule long-read sequencing technology enabling long sequencing reads (up to 60 kb) have been developed for use in transcriptome sequencing [9]. The use of such long-read isoform sequencing (Iso-Seq) offers a more complete characterization of full-length transcriptomes, due to Iso-seq's ability to completely sequence both 5′ and 3′ UTRs and the polyA tails of cDNA molecules [10]. Nevertheless, long-read sequencing has its own limitations such as inability to accurately quantify gene expression, due to the relatively lower throughput of long-read platforms compared to short-read methods.

Long-read sequencing has been previously used to study the full-length transcriptomes of other caner types. Analysis of SK-BR-3 breast cancer cells by genome and PacBio transcriptome sequencing led to the characterization of previously undiscovered fusion transcripts, copy-number amplifications, and structural variants [11]. Another study used long-read Iso-seq and short-read RNA-seq in wild-type and paclitaxel-resistant MDA-MBA-231 sublines (another breast cancer cell line) to identify novel targets of chemotherapy resistance [12]. Full-length transcriptomes can also be studied with Oxford Nanopore sequencing, and full-length transcripts have been sequenced from lung cancer cell lines to identify transcript variants and mutations using the MinION sequencer (Oxford Nanopore Technologies) [13].

In this study, we combined the Iso-Seq technology with short-read RNA-seq methods to survey the full-length GC transcriptome landscape. We selected cell lines from different GC subtypes for RNA sequencing using Iso-seq protocols to systematically characterize the complexity of GC isoforms. We then quantified the expression levels of these isoforms using conventional short-read data in GC cell lines and primary samples. Our results provide the first full-length GC transcriptome database, which will likely prove useful to further understand the molecular basis of GC tumorigenesis and for identifying novel biomarkers and drug targets.

## Results

### Landscape of long-read full-length isoforms in GC cell lines

**Fig. 1:**
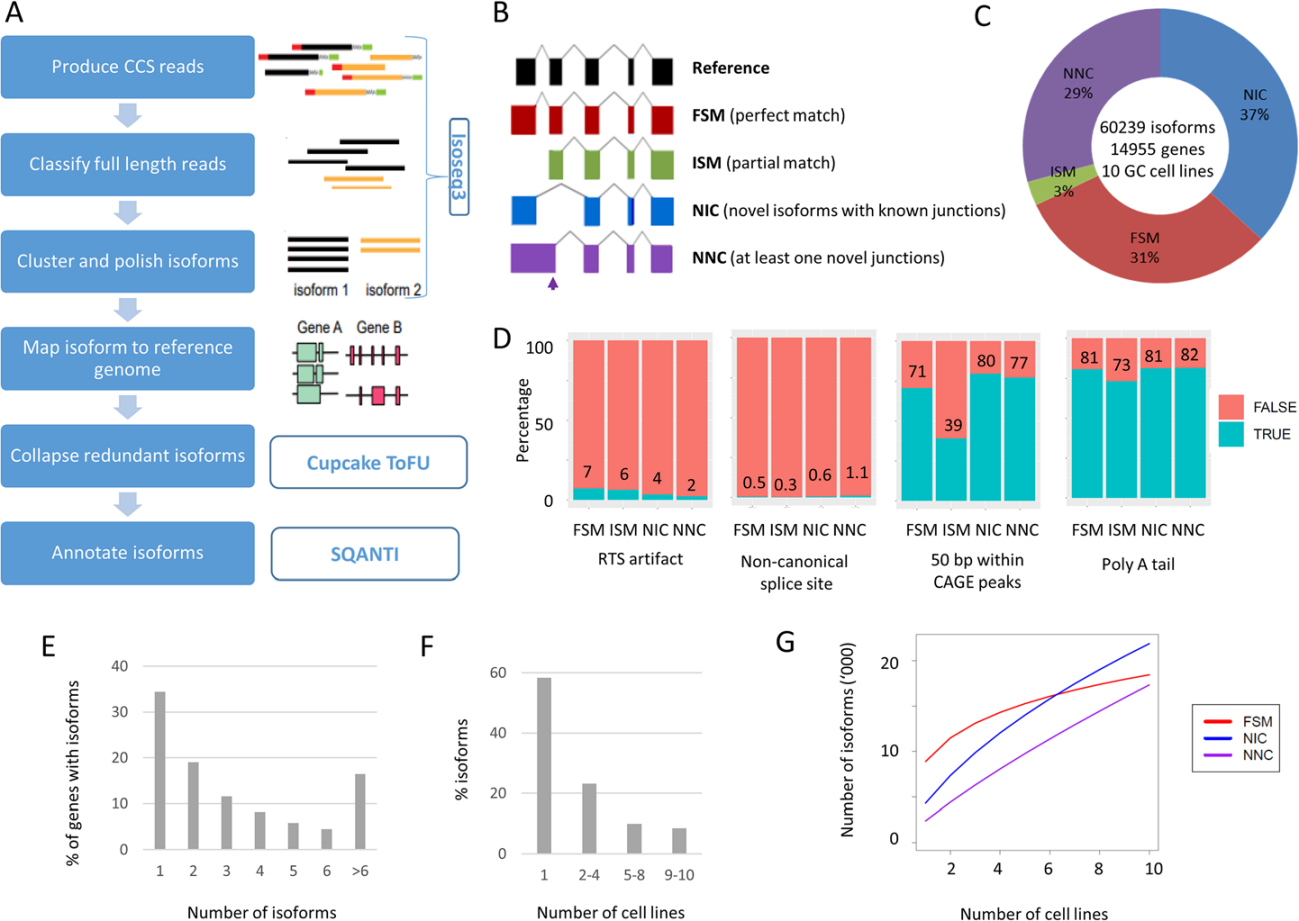
Landscape of long-read transcriptome in gastric cancer cell lines. **a** Isoform calling algorithm for Iso-seq data. **b** Types and illustration of identified isoforms. **c** Breakdown of isoforms identified. **d** Quality control metric for the predicted isoforms. Compared to high-confidence FSM transcripts, NIC and NNC have similar quality metric. ISM has low percentage of transcripts with CAGE support, suggesting some of these transcripts are due to 5′ degradation. **e** Number of isoforms per gene. **f** Number of cell lines per isoforms. **g** Number of isoforms detected vs number of cell lines profiled. Known FSM transcripts (red) are reaching saturation, while novel transcripts are continually discovered

To obtain a representative overview of full-length transcripts in GC, we performed PacBio long-read RNA sequencing on ten GC cell lines. The GC lines were selected to represent four TCGA GC subtypes (CIN—chromosomal unstable, EBV—Epstein-Barr virus positive, GS—genome stable, and MSI—microsatellite unstable) based on previous literature and in-house molecular analysis [5] (Additional file 2, Table S1). For each line, we generated ~ 26 Gb of raw sequencing data, and used the circular consensus sequence module of the IsoSeq3 program (https://github.com/PacificBiosciences/IsoSeq3) to generate consensus reads (Fig. 1a). The consensus reads were filtered for full-length non-chimeric (FLNC) reads. To identify unique isoforms, the FLNC reads were subjected to de novo clustering using the IsoSeq3cluster module. All isoforms were mapped to the human genome (version hg38) using GMAP [14], and only high-quality isoforms (supported by at least two FLNC reads) were considered for further analysis. On average, we identified ~ 37,700 non-redundant full-length isoforms per cell line. Further quality control and isoform annotations were performed using SQANTI2 (https://github.com/Magdoll/SQANTI2), yielding an average of ~ 27,000 annotated isoforms per line. These outputs are comparable with previously reported human cell line Iso-seq data [12, 15].

In total, we identified 60,239 non-redundant transcript isoforms across the ten GC lines and classified the isoforms into four groups based on the Gencode v32 human reference transcriptome database (Fig. 1b provides an illustrative cartoon). Among the isoforms, 31% (18442) were full-splice matches (FSM) matching perfectly to known transcripts, and 37% (21874), 29% (17333), and 3% (1709) were novel in catalog (NIC; corresponding to isoforms with at least one unannotated splice site), novel not in catalog (NNC; corresponding to isoforms with known splice sites but novel splice junctions), and incomplete-splice matches (ISM; corresponding to isoforms that match to a subsection of a known transcript) (Fig. 1c). We used various quality features provided by SQANTI2 to assess the reliability of the full-length isoforms, including non-canonical junction usage, intrinsic sequencing properties (i.e., number of predicted reverse transcriptase template switching artifacts), and functional genomic evidence such as overlap of 5′ transcript ends with independently published Cap Analysis of Gene Expression (CAGE) data [16] (CAGE comprises tag sequencing data directly measuring the 5′ end of transcripts), and 3′ ends with polyA tails (Fig. 1d). Benchmarking the novel isoforms against high-quality known isoforms (FSMs), we found that NIC and NNC novel isoforms exhibited comparable quality to known isoforms, while ISMs exhibited a lower proportion of overlap with CAGE peaks. It is possible that some ISM isoforms may comprise partial fragments resulting from incomplete retro-transcription or mRNA decay artifacts [17]. Due to this concern, we therefore excluded the ISM isoforms (3%) from downstream analysis.

Besides the FSM, NNC, NIC, and ISM isoform categories, SQANTI2 also generates small numbers of transcripts classified as antisense (*n* = 261; 0.4%), genic (*n* = 304; 0.5%; isoforms that overlap with intron), and intergenic (*n* = 316; 0.5%; isoform in intergenic regions) (see Additional File 2; Table S2). Previous studies have indicated that these isoforms tend to be single-exonic with higher percentages of non-canonical splice junctions, which may be caused by experimental or technical artifacts [17]. Due to these reasons and the small numbers of isoforms in these categories (< 1%), we did not consider these categories (antisense, genic, intergenic) and our study only focuses on alternative splicing events found in the FSM, NIC, and NNC categories (*n* = 57,649).

The transcript isoforms (*n* = 57,649) mapped to more than 14 K genes (*n* = 14,203), with 67% of genes associated with > 1 isoform (9462 genes). Each gene was associated with a median of 2 isoforms (Fig. 1e). The majority of isoforms (33,271, 58%) were expressed in only one cell line, and 3513 isoforms (6%) were found in all cell lines (Fig. 1f). Interestingly, rarefaction curve analysis revealed that while discovery of known isoforms experienced saturation, discovery of novel isoforms remained unsaturated (Fig. 1g). To assess the discovery of isoforms as a function of sequencing depth, we also performed rarefaction analysis in each individual cell line by subsampling the number of full-length reads. As shown in Additional File 1; Figure S1, we found that for each cell line, at a sequencing depth of 26 Gb, the discovery of isoforms reached saturation. Thus, the increase in novel isoforms across cell lines is more likely attributable to cell line-specific transcriptomes rather than a lack of coverage in the individual cell lines. These analyses suggest that interrogating the transcriptomic landscape of novel isoforms remains a rich area of untapped biological diversity.

To explore relationships between alternative splicing events and somatic alterations, we integrated the alternative splicing data with somatic mutations identified by whole exome sequencing of the 10 lines. Somatic mutations were identified using Mutect2 [18] on tumor-only mode, with germline subtraction using the gnomAD database [19] and a panel of 36 normal exome samples. Somatic changes were further annotated using Funcotator, and all variants classified as Splice Site were inspected. This analysis identified a total of 335 splice site mutations in the 10 lines. Manual inspection of these mutations in IGV highlighted 49 of these mutations which may lead to changes in splicing at the mutated exons, as detected from the Iso-seq data. This suggests that the vast majority of the splicing alterations identified (NIC and NNC isoforms; *n* = 39,207 compared to 49) are due to transcriptional deregulation rather than somatic alterations.

### Characteristics of long-read novel isoforms

**Fig. 2:**
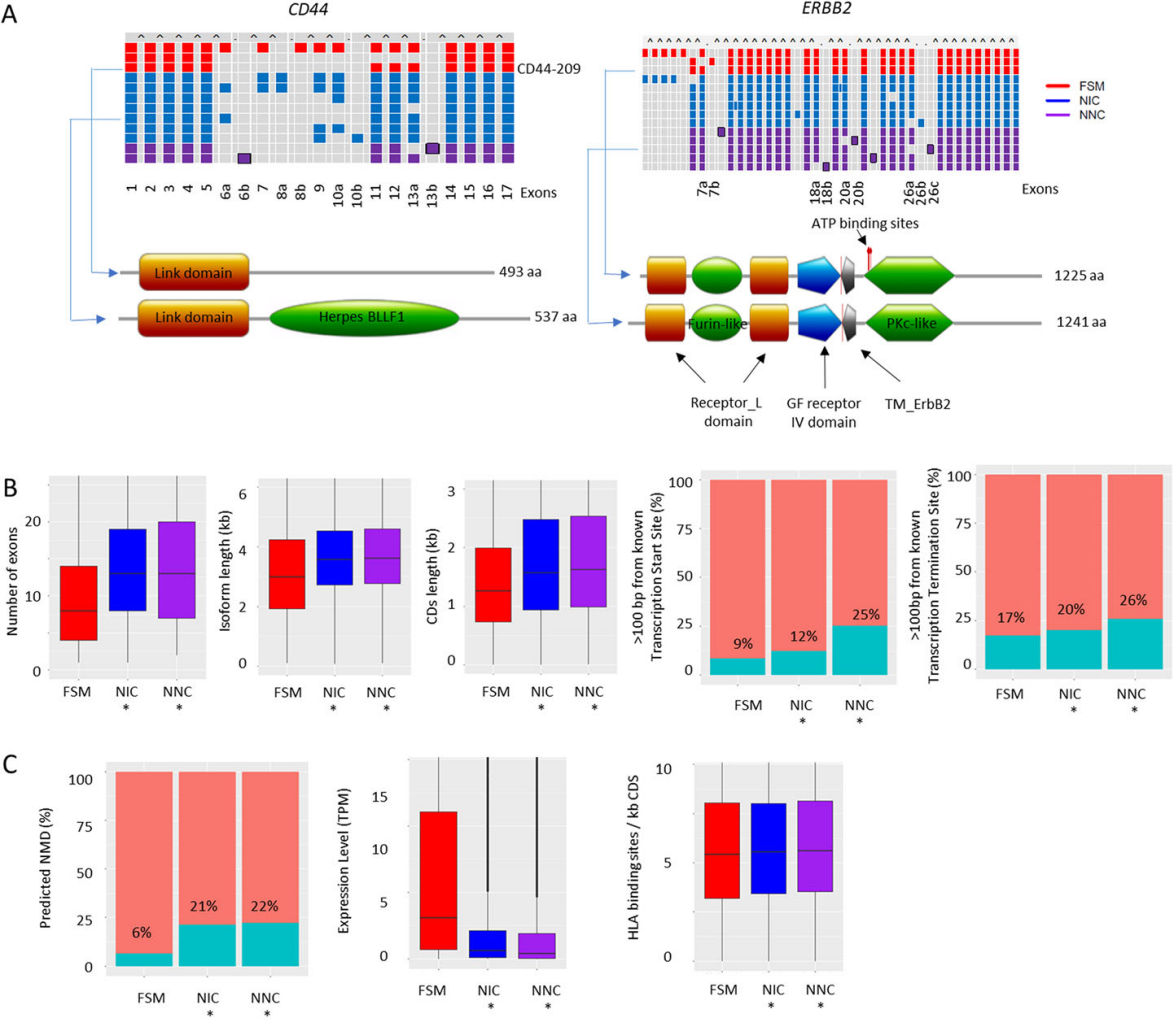
Characteristics of Iso-seq transcripts. **a** Examples of alternative splicing (*CD44*) and alternative promoter (*ERBB2*) events identified in the Iso-seq data. Exon orders are labeled at the bottom, and overlapping exons are indicated by alphabets. For *ERBB2*, only overlapping exons are labeled. Novel exons (relative to Gencode annotation) were indicated in black. Protein domain annotation for selected isoforms was shown in the lower panel. **b** Characteristics of novel (NIC and NNC) and known (FSM) transcripts. NIC and NNC have more exons and longer isoforms and CDS. Novel transcripts can generate novel TSS and TTS. **c** Novel isoforms are more likely to be target of NMD, expressed at lower level and contain more MHC binding sites than known isoforms

We proceeded to characterize the novel transcript isoforms. Of 39,207 novel isoforms, 17,333 (44%) were classified as NNC and the remaining novel isoforms were NIC. We observed NNC and NIC isoforms involving cancer-associated genes such as *ERBB2* and *CD44* and confirmed previously known FSM isoforms associated with these two genes (Fig. 2a). For example, we identified an NNC *ERBB2* isoform with an alternative 3′ exon splice site in exon 26. This splicing event is predicted to cause the loss of 14 amino acids partially deleting the *ERBB2* tyrosine kinase domain. As another example, we identified a *CD44* NIC isoform resembling the known CD44-209 variant isoform but with an additional exon 6a, resulting in gain of a Herpes_BLLF1-like domain (CD-search, e-value = 2.3 × 10^− 3^).

Compared to known isoforms, novel transcript isoforms (both NNC and NIC) possessed larger numbers of exons (median 13 vs 8, Wilcoxon test, *p* value < 2.2 × 10^− 16^), longer transcript lengths (median 3593 vs 2986.5 bp, Wilcoxon test, *p* value < 2.2 × 10^− 16^), and protein coding sequences (median 1593 vs 1260 bp, Wilcoxon test, *p* value < 2.2 × 10^− 16^) (Fig. 2b). Novel isoforms were also more likely acquire new transcription start sites (TSSs) (11% isoforms more than 1 kb away from known TSSs vs 1%; Fisher test *p* value < 2.2 × 10^− 16^) and termination sites (TTSs) (14% isoforms more than 1 kb away from known TTSs vs 8%; Fisher test *p* value < 2.2 × 10^− 16^). Use of new termination sites was also associated with a higher likelihood of premature stop codons associated with nonsense-mediated mRNA decay (NMD) (22% vs 7%; Fisher test *p* value < 2.2 × 10^− 16^). We then used Kallisto [20] to infer the expression levels of the full-length isoform from short-read RNA-seq data and netMHCpan [21] to identify potential antigenic peptides from the annotated isoforms. Novel isoforms were also expressed at lower levels compared to known isoforms (median TPM 0.54 vs 3.08; Wilcoxon test, *p* value < 2.2 × 10^− 16^) and contained a higher proportion of major histocompatibility complex (MHC) binding sites (median 5.6 binding site per kb vs 5.4; Wilcoxon test, *p* value 9.8 × 10^− 7^) (Fig. 2c). Further studies are required to determine if these predicted MHC binding affinity differences are associated with biologically relevant patterns of immunogenicity and T cell responses.

We next compared transcriptome and expression profile levels generated using either long-read Iso-seq or short-read RNA-seq methods. We performed isoform discovery on Illumina short-read RNA-seq using Stringtie (reference-guided and allowing for novel isoforms) in the same ten cell lines profiled by Iso-seq. Similar to the Iso-seq analysis, identified isoforms were annotated using SQANTI2 and grouped according to FSM, NIC, and NNC categories. Using gffcompare, we compared the long-read and short-read methods and found that most isoforms identified from long reads (66.8%) could not be readily recovered from short-read data alone (Additional File 1; Figure S2A). Moreover, although the short-read-only analysis identified larger numbers of isoforms, further examination revealed that compared to the long-read analysis, the short-read data had much shorter isoform lengths (2182 bp vs 3564 bp, *t*-test *p* value < 2.2 × 10^− 16^), contained less exons (7.2 vs 13.1, *t*-test *p* value < 2.2 × 10^− 16^), and were less strongly supported by CAGE (38.3% vs 70.3%, Fisher test *p* value < 2.2 × 10^− 16^) and polyA data (45.0% vs 81.3%, Fisher test *p* value < 2.2 × 10^− 16^). These results suggest that many predicted short-read isoforms are incomplete fragments of full-length isoforms, rather than true isoforms (see Additional File 1; Figure S2B, C).

We then compared the use of Iso-seq and RNA-seq data to estimate isoform expression, focusing on the subset of isoforms identified using both methods (*n* = 19,094). We calculated isoform expression levels (tpm) using

1. Iso-seq data only (FL-TPM; full-length read transcript per million)
2. RNA-seq data using Iso-seq-defined transcriptomes (TPM; kallisto)
3. RNA-seq data using short-read-defined transcriptomes (TPM; kallisto)

This analysis revealed that Iso-seq-derived expression level are only weakly correlated to the RNA-seq-derived expression levels (Pearson *r* = 0.27, Spearman *r* = 0.5), which is likely due to the relatively lower depth of sequencing in Iso-seq. However, transcript expression levels using RNA-seq reads mapped onto Iso-seq or short-read transcriptomes were highly correlated (Pearson *r* = 0.92, Spearman *r* = 0.87) (see Additional File 1; Figure S2D). Taken together, these analyses demonstrate that Iso-seq and RNA-seq data are complementary—the use of Iso-seq allows identification of full-length cell line-specific isoforms and GC transcriptomes minimizing assembly errors and artifacts due to incomplete fragments, while the use of high-coverage short-read RNA-seq enables a more accurate assessment of gene expression levels.

**Fig. 3:**
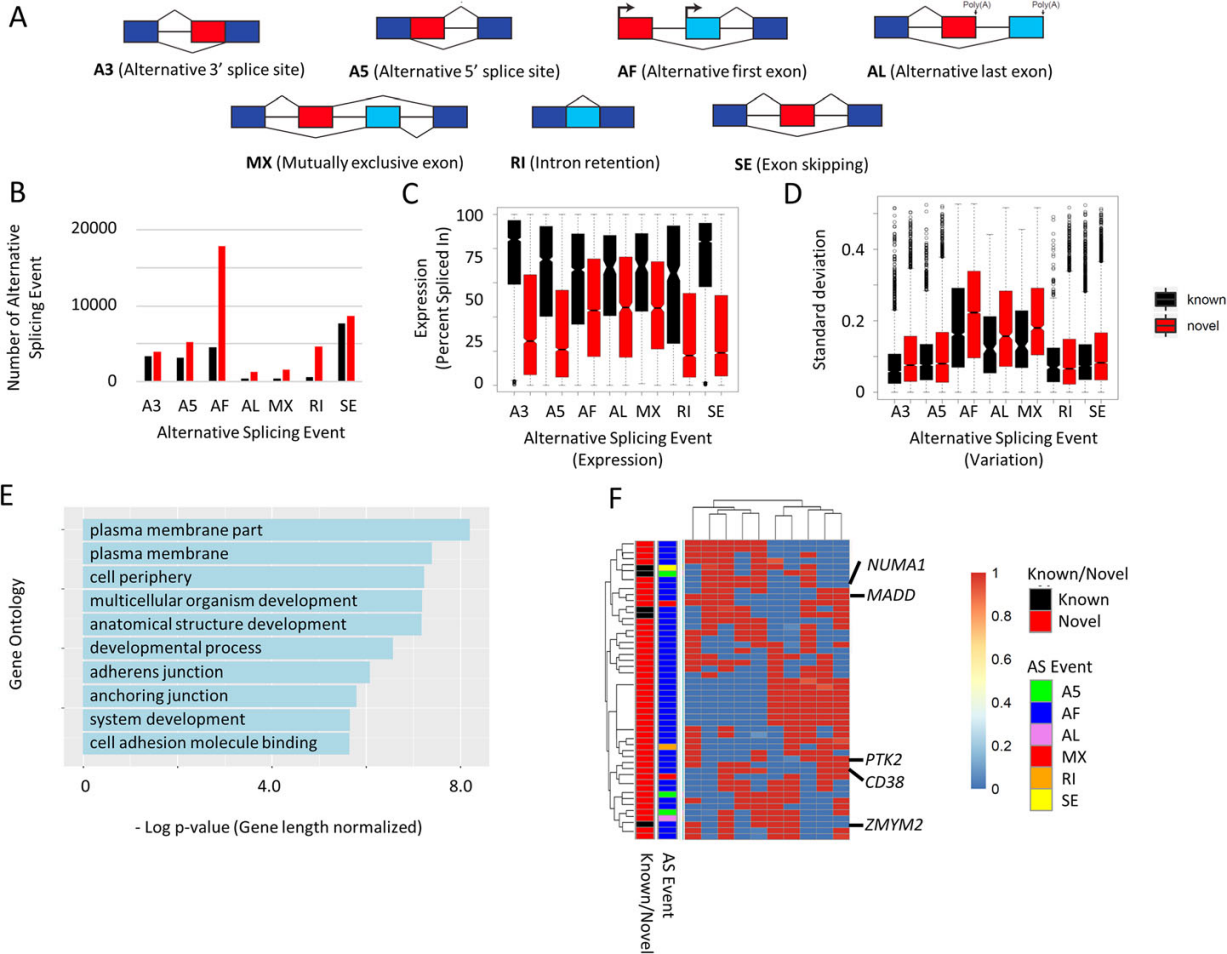
Types of alternative splicing alterations. **a** Classification of alternative splicing (AS) events using SUPPA. A3—Alternative 3′ Splice Site; A5—Alternative 5′ Splice Site; AF—Alternative First Exon; AL—Alternative Last Exon; MX—Mutually Exclusive Exon; RI—Retained Intron; SE—Skipping Exon. **b** Alternative promoter sites (AF) is the most common type of splicing events detected and most AF events are found in novel isoforms (NIC or NNC). **c** Alternative splicing events in novel isoforms are expressed at lower level. Novel AF, AL, and MX show relatively higher expression. **d** Variability of SE event percent spliced in (PSI) observed across SE types. The highest variation is observed in the AF of novel isoforms. **e** Gene ontology analysis of the top 500 most variable SE events—genes involved in commonly dysregulated pathways in gastric cancer are also target of alternative splicing/promoter (such as cell adhesion and developmental processes). *p* value has been adjusted for gene length bias. **f** Heatmap showing the 50 most variable AS events—most of the most variable AS are AF

To further characterize the long-read transcripts, we compared both the known and novel isoforms across different types of splicing events. Using the SUPPA2 tool [22], we quantified levels of intron retention (RI), exon skipping (SE), alternative 3′-acceptor (A3), alternative 5′-donor (A5), alternative first exon (AF), alternative last exon (AL), and mutually exclusive exon (MX) splicing events (Fig. 3a). In total, 63,786 alternative splicing events were identified (Fig. 3b), to which AF events contributed to the greatest degree (22,399, 35.1%). AF events were more likely to be found in novel isoforms compared to known isoforms (Fisher test, *p* value < 2.2 × 10–^16^, odds ratio = 2.4), suggesting that the use of alternative first exons may have been significantly underestimated in previous studies using short-read RNA-sequence data. These data also support previous reports that alternative promoter usage is a major source of transcriptomic and functional diversity in cancer [8].

We also used SUPPA2 to calculate relative expression levels (represented by “percent spliced in”; PSI) and variation in expression for the different splicing classes. Across the GC lines, novel isoforms of every splicing class exhibited lower expression levels compared to known isoforms (average PSI 0.38 vs 0.69 In known isoforms; paired *t*-test *p* value 6.4 × 10^− 4^). However, among the splicing classes, novel AF, AL, and MX (average PSI 0.46–0.47) events exhibited relatively higher expression than other splicing event types (average PSI 0.31–0.37; *t*-test *p* value 1.4 × 10^− 3^) (Fig. 3c). Novel isoforms tended to be expressed more variably (average standard deviation 0.15 vs 0.12; paired *t*-test *p* value 1.5 × 10^− 3^), with AF, AL, and MX events showing the highest variance across lines (average standard deviation 0.20 vs 0.11; *t*-test *p* value 0.01) (Fig. 3d). Hierarchical clustering across the lines revealed that the most highly variable isoforms are often novel isoforms associated with AF events. These findings were robust even when the top 500–1000 most variable splicing events were analyzed (data not shown). Gene ontology analysis of isoforms exhibiting the top 1000 splice events with the highest variance revealed that these isoforms are enriched for pathways known to be deregulated in GC, such as developmental processes and cell adhesion (gene length bias-adjusted *p* values 4.9 × 10^− 8^ and 8.6 × 10^− 6^) (Fig. 3e), including several known cancer genes such as *MADD*, *PTK2*, and *NUMA1* (Fig. 3f).

### Long-read transcriptomes inform analysis of primary GC RNA-seq profiles

**Fig. 4:**
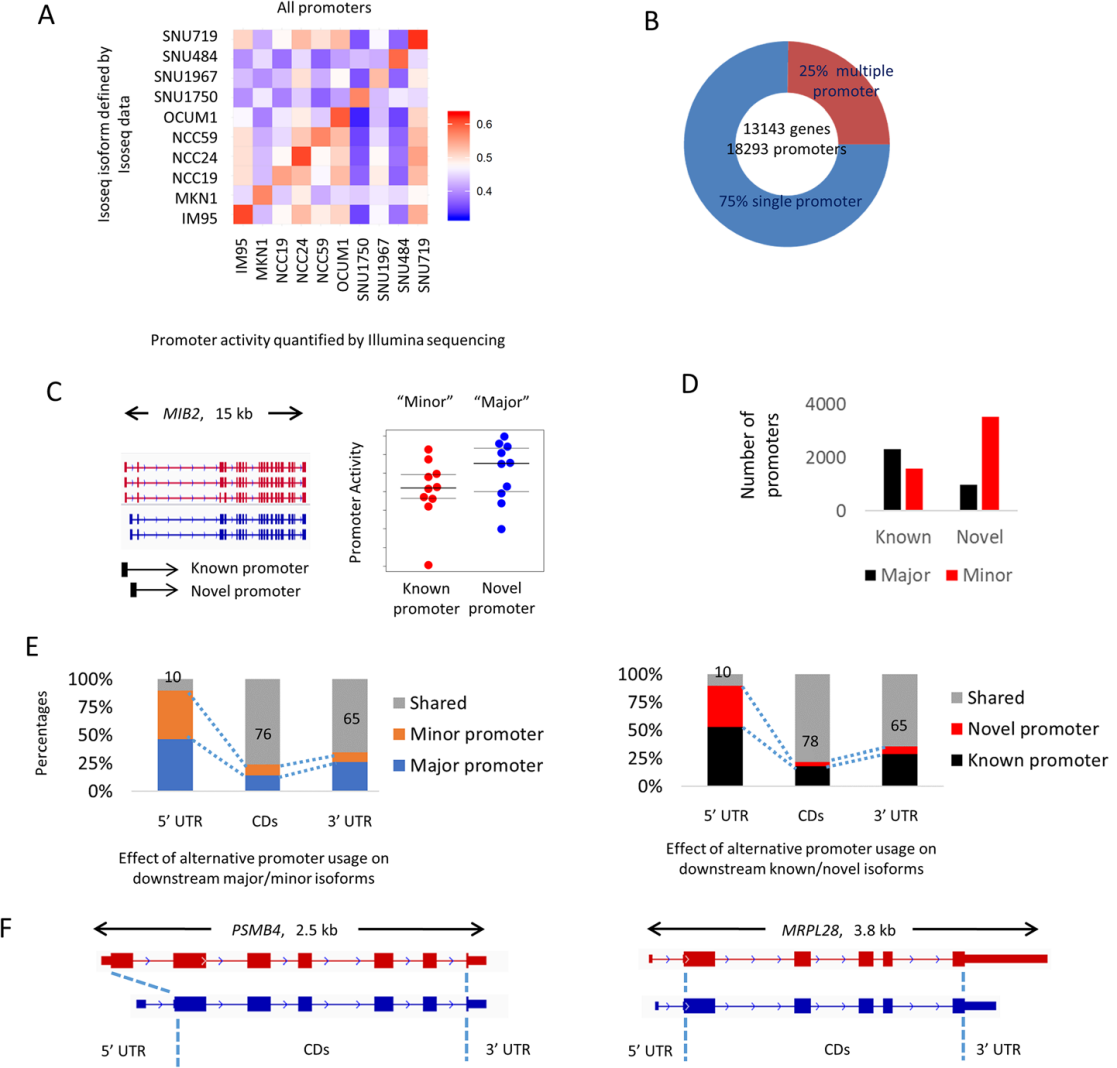
Landscape of alternative promoter usage in the Iso-seq data. **a** Correlation matrix between the detected isoforms from Iso-seq data with the promoter activity estimated from Illumina short-read RNA-seq for isoforms with length less than 2 kb. The highest correlation coefficients are always observed between the predicted promoter activity from the Iso-seq and Illumina data from the same cell line. **b** Number of identified Iso-seq genes and promoters. Twenty-five percent of all genes have multiple promoters. **c** Example of known and novel promoters. Promoters are considered known if at least one FSM transcript is initiated from the promoter site, and novel if no FSM transcript is initiated from the promoter site. Gene promoter with the higher average activity is additionally assigned as major promoter and all other promoters for the same gene are assigned as minor promoters. **d** Assignment of major and minor promoter from short-read RNA-seq data. Novel promoters are often minor promoters. **e** Schematic representation of the relationship between 5′ UTR, CDs, and 3′ UTR regions. On average, usage of alternative promoters modifies about 22–24% of coding regions. **f** Example of how alternative promoter usage can modify CDs (*PSMB4*) and 5′ UTR (*MRPL28*)

Given the enrichment of alternate promoters in novel isoforms and their high inter-sample variability, we focused in depth on this specific splicing sub-class. Here, we applied *proActiv* [8], an R package that estimates promoter activity from aligned RNA-seq data applied onto a reference transcriptome. Briefly, *proActiv* quantifies promoter expression using a set of unique junction reads, and we have previously shown that promoter activity predicted by *proActiv* shows higher consistency with CAGE and H3K4me3 histone data when benchmarked to other methods. To evaluate the accuracy of the *proActiv* promoter predictions, we correlated predicted promoter activities from standard RNA-seq with the predicted transcriptomes derived from Iso-seq at different ranges of isoform lengths (Additional file 2, Table S3). We observed stronger correlations for shorter isoforms compared to longer isoforms. Importantly however, at all isoform length categories, we observed significantly higher correlations between promoter activities inferred from Iso-seq and *proActiv* software in the same cell line, compared to promoter activities inferred from different cell lines, suggesting that promoter activity inferred using *proActiv* is most consistent with the Iso-seq data from the same cell line. For example, for isoforms with gene length less than 2 kb, the average correlation coefficient between the same lines was 0.63, compared to 0.49 when compared between different cell lines (Fig. 4a; *t*-test *p* value 1.8 × 10^− 12^). Similar correlations were observed when restricting this analysis to either only known or novel isoforms (Additional file 2, Table S4). The moderate correlation observed between Iso-seq and *proActiv* from the same cell lines is likely due to the relatively lower sequencing depth and gene length biases in Iso-seq methods. The approach of using the Iso-seq full-length isoforms to generate a reference transcriptome and subsequently quantifying the isoform expression using short-read has also been used by others in the field [23–26].

Across the GC lines, we identified 18,293 active promoters mapping to 13,143 genes (Fig. 4b). Twenty-five percent of genes (3257) were associated with at least 2 distinct promoters. We classified the active promoters as major and minor depending on their average promoter activity in the 10 cell lines, and also classified them as known or novel promoters (Fig. 4c). We found that promoters associated with novel isoforms are often minor promoters (Fisher test, *p* value < 2.2 × 10^− 16^) which are expressed at lower levels (Fig. 4d). However, 21% of novel isoforms were predicted to be the major promoter in the GC lines (e.g., *MIB2*, see Fig. 4c). To understand the consequence of alternative promoter usage on downstream functional regions, we calculated the shared fraction of 5′ untranslated regions (UTRs), coding sequence regions (CDs), and 3′UTRs between isoforms initiated by distinct promoters. We found that changes in 5′UTR regions are often followed by changes in downstream CDs and 3′UTRs, with the majority of known/novel promoter pairs exhibiting potential changes in their CD composition (1734/2059; 84.2%). The average extent of changes observed per promoter pair (major/minor or known/novel promoters) was 22–24% for CDs and 35% for 3′UTRs (Fig. 4e). Figure 4f shows examples of alternative promoter usage associated with downstream usage of distinct CDs and 3′ UTR regions.

To validate expression of the novel isoforms at the protein level, we queried an in-house mass spectrometry proteomics dataset of the 10 cell lines analyzed by Iso-seq. Briefly, GeneMarkS-T predicted protein coding sequences for all isoforms were added to the Gencode v32 protein-coding sequence database to form a reference proteome. Unique peptides were identified using MaxQuant [27] with the use of this reference proteome. This proteomic analysis identified 930 unique peptides from 428 Iso-seq proteins that are not found in the Gencode v32 database (Additional File 2; Table S5). Importantly, we were able to validate several unique peptide sequences associated with novel promoter sites (Additional File 1; Figure S3), supporting the idea that many novel Iso-seq isoforms are indeed expressed at the peptide level. In addition, we also performed 5′ Rapid Amplification of cDNA Ends (RACE) to validate two novel isoforms (*FGFR4*, *TMEM59*; Additional File 1; Figure S4a), and Western blotting validation of novel ARID1A and TMEM59 isoforms (Additional File 1; Figure S4b). Notably, 5′ RACE and protein expression of the novel MET isoform has been previously reported [28–30], further validating the ability of our pipeline to identify novel cancer-associated promoters. These results suggest that alternative promoter usage may contribute to functional diversification of the proteome by allowing for a single gene to select for multiple protein-coding sequences.

**Fig. 5:**
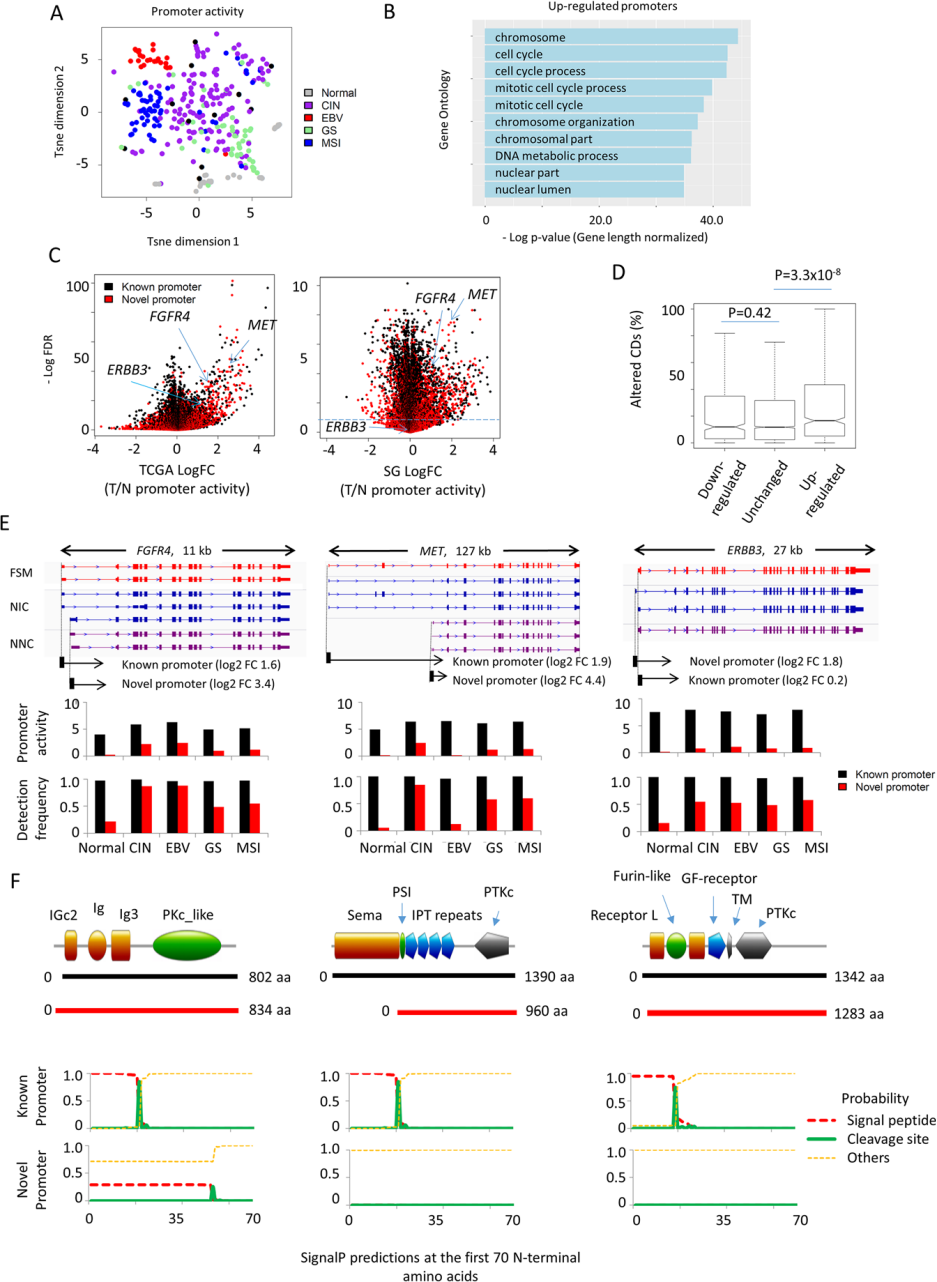
Quantification of Iso-seq transcriptome in TCGA gastric cancer dataset. **a** T-sne plot of promoter activity in 315 TCGA gastric cancer samples (282 tumors and 33 normal). **b** Gene ontology analysis of the upregulated promoters in TCGA GC dataset. *p* value has been adjusted for gene length bias. **c** Volcano plot showing the log fold change in promoter activity in tumor samples. Novel promoters of several gastric cancer oncogene (*FGFR4*, *MET*, and *ERBB3*) are upregulated in the TCGA dataset (right). In an independent dataset (20 T-N pairs), novel promoters of *MET* and *FGFR4* are also found to be upregulated while novel promoter for *ERBB3* is not significantly upregulated. **d** Altered CDs by promoter activity status. Upregulated promoters have larger CD alteration. **e** Upper panel shows the gene isoforms initiated from the known and novel promoters of *FGFR4*, *MET*, and *ERBB3*. Lower panel shows the promoter activity and detection frequency in normal samples and TCGA subtypes of gastric cancer. Novel promoters are expressed at a lower level, but exhibited higher upregulation and are expressed in more tumor samples compared to normal samples. **f** Upper panel shows the protein domains of isoforms initiated from the known and novel promoters of *FGFR4*, *MET*, and *ERBB3*. Lower panel shows the signal peptide prediction in transcripts initiated from known and novel promoter sites, showing loss of signal peptide sequences in the novel isoforms

Using *proActiv*, we then extended our full-length transcript-informed promoter predictions to the TCGA GC RNA-seq dataset (282 gastric cancer and 33 normal samples). We observed that promoter activity is distinct between tumor and normal samples, and also between different molecular subtypes of GC (Fig. 5a). We then applied DESeq2 to perform differential promoter usage analysis on the tumor and normal samples. Comparison between tumor and normal samples revealed 2389 upregulated and 2049 downregulated isoforms in GC (FDR < 1 × 10^− 3^; Additional file 2, Tables S6 and S7). Notably, promoters upregulated in GC (*n* = 2389) were significantly more likely to have changes in their CDs (average extent of altered CDs per promoter pair, 27.5% vs 20.9%; *t*-test *p* value 3.3 × 10^− 8^) and associated with cancer-related gene ontologies such as chromosomal organization (gene length-adjusted *p* value 5.4 × 10^− 38^) and cell cycle (gene length-adjusted *p* value 3.2 × 10^− 43^) (Fig. 5b). We observed upregulated isoforms comprising novel promoter isoforms of known oncogenes, such as *MET*, *FGFR4*, and *ERBB3* (Fig. 5c, right). Repeating this analysis in a second independent cohort of 20 pairs of gastric cancer samples re-identified *MET* and *FGFR4* as upregulated in GC samples (Fig. 5c, left). Downregulated promoters were not associated with CD changes (21.8% vs 20.9%; *t*-test *p* value 0.42) (Fig. 5d).

The three novel *MET*, *FGFR4*, and *ERBB3* isoforms were predicted to be initiated from promoters distinct from their annotated TSSs (51 kb away (*MET*), 438 bp (*FGFR4*), 248 bp (*ERBB3*), and upregulated in tumor samples to a greater degree from their known isoforms (Fig. 5e). Similar to the overall population, promoter activities and detection frequencies (a surrogate of expression abundance) were lower for these novel isoforms compared to known isoforms, and the novel isoforms exhibited greater between-tumor variability. For example, the novel *MET* isoform is highly expressed in the CIN subtype (log2 fold change = 2.1, *p* value = 5.5 × 10^− 16^), but depleted in EBV (log2 fold change = − 4.7, *p* value = 9.4 × 10^− 15^) compared to other GC samples. Comparisons of CDs revealed that most functionally important protein domains are retained in the novel isoforms; however, all three novel isoforms showed N-terminal protein truncations and removal of the Sema domain in the case of *MET* (Fig. 5f)*.* All three novel isoforms initiated from the new promoter sites are predicted to disrupt signal peptide sequences required for localization to the cell membrane. Similar mechanisms had been reported whereby alternative promoter usage leads to protein localization at different cellular compartment [31]. Taken together, these observations suggest that novel promoter sites may allow genes to acquire new functional roles and be regulated in a subtype-specific manner.

We next queried ReMap [32] to identify TFs enriched at tumor-specific promoters. The ReMap 2018 atlas contains transcriptional regulator peaks derived from curated ChIP-seq, ChIP-exo DAP-seq, and ENCODE databases extracted from GEO (Gene Expression Omnibus) comprising 485 transcription regulators across 346 human cell types from 2829 ChIP-seq datasets. We integrated the 485 TF occupancy profiles against significantly upregulated alternative promoters (FDR < 0.001; *n* = 2389) compared to all promoters identified in this study (*n* = 18,293) using the ReMapEnrich R package (https://github.com/remap-cisreg/ReMapEnrich). From 485 TFs available in Remap, 204 TFs were found to be significantly increased (*q* < 0.001) at the upregulated promoters in at least one ChIP-seq experiment. To assess the robustness of our analysis, we also performed the same analysis using a different TF-DNA direct interaction database (UniBind). UniBind contains information on 231 TFs from 1983 ChIP-seq datasets. Four of the 10 highest-ranked TFs by ReMap were also predicted by the UniBind_enrichment tool (https://unibind.uio.no/enrichment/), including E2F4, E2F1, MYC, and MXI1 (Additional File 1; Figure S5a, S5b). These may highlight possible TFs regulating the use of alternative promoters in GC.

We also examined the promoters for changes in DNA methylation, using genome-wide MeDIP-seq (methylation-dependent immunoprecipitation followed by sequencing) for 9/10 cell lines. Briefly, MeDIP reads were aligned to the human genome using bwa, and duplicates removed using samtools. DNA methylation peaks were called using MACS2 with input control. In 7/9 cell lines, we observed that non-expressing isoforms (as measured using Iso-seq data) tended to have higher methylation levels near their promoter region. In contrast, expressed isoforms tended to have reduced DNA methylation levels—this correlation was observed for both known and novel promoters (Additional File 1; Figure S6), providing further evidence that the novel expressed promoters are *bona-fide* promoters as they exhibit similar epigenetic features to known expressed promoters.

### Clinical outcome associated with alternative promoter usage

**Fig. 6:**
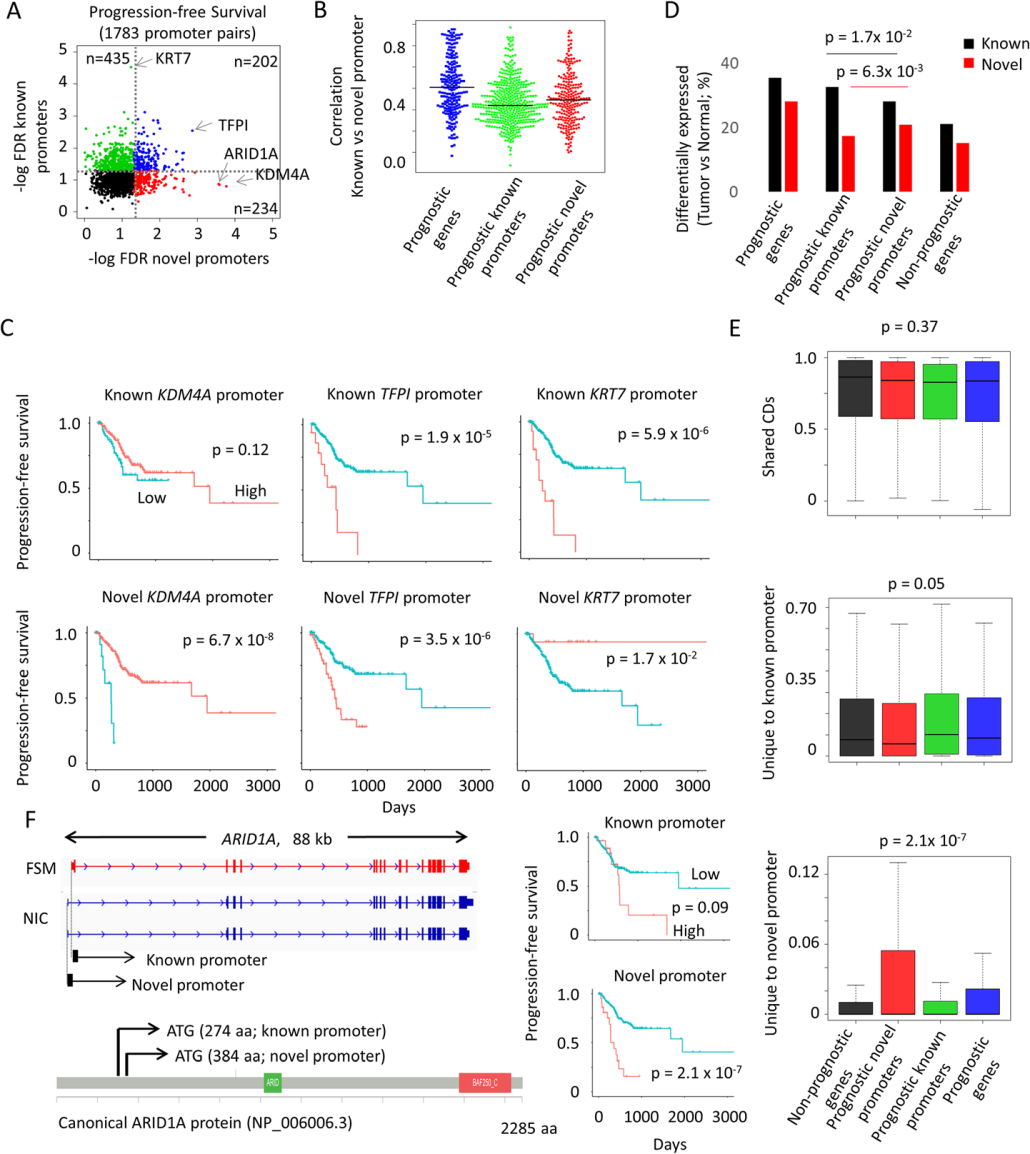
Clinical outcome associated with alternative promoter usage. **a** Scatterplot of adjusted *p* values for genes with known and novel promoters. Examples of prognostic gene (in blue), prognostic known promoter (in green), and prognostic novel promoters (in red) include *TFPI*, *KRT7*, and *KDM4A*, respectively. **b** Beeswarm plot showing the average correlation between known and novel promoters. There are more prognostic promoters (known or novel) than prognostic genes and prognostic promoters have lower correlation than prognostic genes, suggesting distinct promoters are independently regulated. **c** Survival plots showing significant association of novel and known promoters of *KDM4A*, *TFPI*, and *KRT7* with progression-free survival. **d** Percentages of known and novel promoters that are differentially expressed in prognostic genes, prognostic promoters, and non-prognostic genes. Deregulated expression of genes and isoforms are prognostic of survival. **e** Boxplots of the shared and private CD percentage in prognostic genes, prognostic promoters, and non-prognostic genes. Prognostic promoters are enriched with promoter-specific CD regions. **f** Gene structure of the known and novel *ARID1A* promoters at the transcript and protein level. Survival curve shows the association between the known and novel promoters of *ARID1A* with progression-free survival in gastric cancer

Because novel promoters are variably expressed, we explored if their usage might highlight new biomarkers of clinical outcome in GC. To test this possibility, we correlated the different promoter activity patterns to progression-free survival in the TCGA dataset (Fig. 6a). Tumor samples were stratified into high and low promoter usage, based on optimal cutoffs determined using the surv-cutpoint function, and the length of survival in the two groups compared using the log-rank test.

In 1783 genes with predicted known and novel promoters, we have identified 871 genes with prognostic promoters (FDR < 0.05). Of these, 202 genes (23%) were prognostic for both known and novel promoters, and as expected for these genes, promoter activity of the known and novel promoters were highly correlated (average correlation 0.43). In contrast, 435 and 234 genes were prognostic for only either the known or novel promoter respectively, and for these genes, promoter activity of the known and novel promoters was uncoupled (average correlation 0.28, *t*-test, *p* value 1.6 × 10^− 11^) (Fig. 6b). Examples of genes with prognostic known promoters (e.g., *KRT7*), prognostic genes (e.g., *TFPI*) and prognostic novel promoters (e.g., *KDM4A*) are shown in Fig. 6c. Compared to non-prognostic genes, promoters for prognostic genes and promoters themselves are more likely to be differentially expressed in GC (Fisher test, *p* value 1.5 × 10^− 13^, odds ratio = 1.8) (Fig. 6d). Intriguingly, we found that isoform-specific CD regions associated with novel promoter-specific CD regions are specifically enriched for genes with prognostic novel promoters (Kruskal-Wallis test, *p* value 2.1 × 10^− 7^) (Fig. 6e), suggesting that the gain of CDs due to the usage of novel promoter sites may confer genes with additional roles. In contrast, there was a significantly weaker association for isoform-specific CD regions associated with known promoters (Kruskal-Wallis test, *p* value 0.05). This analysis further supports the hypothesis that novel promoters can be independently regulated and that they may have distinct roles from the known counterparts.

As an example, we discovered a novel *ARID1A* isoform that is significantly associated with shorter progression-free survival (Fig. 6f). We identified 3 *ARID1A* transcript isoforms and 2 promoters from the catalog of full-length GC isoforms. The detected novel and known protein isoforms of *ARID1A* are predicted to truncate the first 384 and 274 N-terminal amino acid respectively from the canonical protein (NP_006006.3). Protein isoforms corresponding to the canonical *ARID1A* transcript in Ensembl (the Ensembl transcript with the longest CDs translation) were not detected in our dataset. Interestingly high expression of the novel promoter is associated with poorer survival (log-rank *p* value 2.1 × 10^− 7^) whereas the known promoter is not significantly associated (log-rank *p* value 0.09). The known *ARID1A* promoter is significantly depleted in the MSI subtype (log2 fold change = − 0.35, *p* value = 3.2 × 10^− 3^). In contrast, the novel *ARID1A* is not differentially expressed in MSI (log2 fold change = 0.04, *p* value = 0.88) but shows borderline downregulation (log2 fold change = − 0.42, *p* value 4.2 × 10^− 2^) in the CIN subtype. Further study is required to elucidate the functional roles of the different *ARID1A* isoforms.

## Discussion

GC is a heterogenous disease, with a significant global health burden. Previous transcriptomic studies of GC using short-read RNA sequencing have proved informative in identifying new prognostic biomarkers and drug targets such as fusion genes [33]. One limitation, however, is that short-read RNA sequencing often cannot accurately identify full-length isoforms that are differentially expressed under distinct disease states and between cancer subtypes. Achieving a full molecular understanding of GC therefore requires a comprehensive analysis of alternative splicing events at the isoform level. For example, *BCL2L1* has an alternative 5′ splice site in intron 2 that produces two distinct protein isoforms [34]. One isoform, BCL-X_S_, promotes apoptosis while the other, BCL-X_L_, inhibits apoptosis and is preferentially expressed in cancers [35]. Similarly, inclusion of exon 6 in the FAS receptor *TNR6* produces a membrane-bound receptor isoform that can relay external signal leading to apoptosis [36]. In contrast, *TNR6* isoforms that skip exon 6 do not induce apoptosis and are found in higher concentrations in cancer patients [37, 38].

In this study, we have generated to our knowledge, the first full-length transcriptome database of GC cell lines across different subtypes, employing the PacBio Iso-seq platform. We used this database to study alternative splicing alterations and novel transcript isoforms that are differentially expressed in GC. Supporting the richness of the Iso-seq data, our analysis revealed that the majority of the identified isoforms are novel not being previously reported in the human Gencode reference transcriptome database. Interestingly, while known Gencode isoforms tended to be detected in larger numbers of cell lines, novel isoforms tended to be more cell line-specific. These observations suggest that many GC tissue-specific isoforms remain unexplored. For example, we found in this study an *ERBB2* isoform that lacks crucial ATP binding sites in the tyrosine kinase domain, and a *CD44* isoform that gains a protein domain associated with surface antigens capable of eliciting an immune response.

Recent large-scale studies have studied the importance of alternative splicing in cancer. For example, analyses of 8705 tumor samples in 32 cancer types revealed that alternative splicing in cancer occurs on average 20% more often than in normal tissues [7]. Interestingly, cancer-associated splicing events were reported to generate thousands of tumor-specific isoforms not expressed in non-malignant samples. In addition, other studies have identified alternative splicing as an important mechanism for drug sensitivity or resistance. Specifically, lung cancer patients with *MET* exon 14 skipping are responsive to *MET* inhibitors despite not having other activating alterations [39], while melanoma patients expressing a BRAF isoform lacking exons 4–8, which encodes for the RAS binding domain, exhibit resistance to BRAF inhibitors [40].

By analyzing different categories of alternative splicing events, we found that alternative promoters (represented by alternative first exons; AF) are frequently used in GC, supporting recent studies reporting the widespread use of alternative promoters in multiple cancer types [8]. Beside finding that alternate promoters are prevalent in the novel GC transcript isoforms, our ability to analyze full-length transcript data also revealed that isoforms initiated from distinct promoter sites often also utilize different downstream protein coding sequences and 3′UTR regions. This finding is significant as it may imply the choice of promoter may also influence downstream RNA splicing events, as alluded to in other reports [41]. Importantly, promoters associated with larger downstream coding sequence alterations were more likely to be upregulated in GC compared to downregulated or unaltered promoters, supporting the hypothesis that some promoters may initiate protein products that are positively selected for during cancer evolution.

By applying the promoter prediction algorithm *proActiv* to our full-length GC transcriptome database, we were the able to further profile the expression level of these promoters in adjacent normal stomach samples and primary GC samples from different TCGA subtypes and with different clinical outcomes. Differential expression analysis reveals promoters upregulated or downregulated in GC compared to normal stomach tissues. These upregulated promoters encode for novel isoforms of receptor tyrosine kinases that are activated in diverse cancer types, such as *MET*, *FGFR4*, and *ERBB3*. Interestingly, in all three cases, the choice of alternate promoters abolishes the N-terminal signal peptide required for targeting to cellular membrane in all three novel isoforms. In the case of *MET*, the deletion also encompasses the ligand-binding Sema domain, indicating that this isoform may be activated via a ligand-independent mechanism. Notably, activation of N-terminal truncated isoforms for *MET* and *FGFR4* has been previously reported before in human musculoskeletal [42] and pituitary tumors [43]. These isoforms have distinct transcription start sites from the *MET* and *FGFR4* isoforms reported in this study. Both reported isoforms also lack the signal peptide and reside mainly in the cytoplasm. In pituitary tumors, membrane-anchored wt-*FGFR4* (wild-type *FGFR4*) formed a complex with neural cell adhesion molecule (NCAM) and N-cadherin, imparting sensitivity to FGFR inhibitor treatment. In contrast, the truncated ptd-*FGFR4* (pituitary tumor-derived *FGFR4*) did not associate with NCAM and interfered with N-cadherin signaling to impede cell adhesion [43]. These reports suggest that alternate promoter-initiated novel isoforms (*FGFR4*, *MET*, and *ERBB3*) may be expressed in distinct cellular compartments and exhibit distinct biological properties from the wild-type counterparts.

Clinical outcome analysis of the promoter activity further demonstrates that most alternate promoters are only weakly correlated in expression with other promoters in the same gene, thereby resulting in distinct and promoter-specific associations with progression-free survival. From our clinical outcome analysis, we found that promoters that are prognostic of patient outcome also tended to harbor a larger proportion of promoter-specific coding regions. One notable finding here is a novel isoform of *ARID1A*, whose expression was positively correlated with poorer progression-free survival. *ARID1A* is a well-known tumor suppressor frequently mutated in GC [44], and the loss of its mRNA or protein expression had been suggested to be associated with poorer prognosis [45, 46] although this association had not been universally accepted. Interestingly, certain splice isoforms of *ARID1A* have also been shown to have oncogenic properties in sarcoma [47]. These findings highlight the need to distinguish and accurately quantify the various gene isoforms associated with alternative promoter usage and potentially different biological roles during GC progression.

Our study has several limitations. First, the sequencing depth from Iso-seq method is likely not yet sufficient to cover the full scope of isoform diversity. Thus, our analysis may have missed genes or isoforms expressed at low levels. Second, owing to their relatively lower sequencing coverage and gene length biases, long-read sequencing is at present less reliable in inferring expression levels than short-read RNA-seq. This may explain the lower than expected correlation of promoter usage from the two methods. Despite these limitations, our results show that the Iso-seq method provides useful information that complement conventional short-read RNA-seq methods.

## Conclusions

In summary, we have surveyed the landscape of full-length transcriptome expressed in GC lines. Having observed substantial level splicing events leading to alternative promoters and previously unknown transcript isoforms, our results strongly highlight that full-length transcriptome profiling represents an under-explored area of research which may yield novel biological insights, biomarkers, and drug targets. The transcriptome data described in this study should thus provide useful and invaluable resource to understanding the importance of transcript isoform expression to the cancer research community and translational researchers focusing on GC and other gastrointestinal malignancies.

## Methods

### Cell lines and cell culture

Cell lines were purchased from Japan Health Science Research Resource Bank (IM95, KATOIII, MKN1, NUGC4, and OCUM1) and Korean Cell Line Bank (NCC19, NCC24, NCC59, SNU719, SNU484, SNU1750, and SNU1967). YCC6 and YCC21 cells were gifts from Yonsei University College of Medicine, Seoul. Cell lines were authenticated using Short Tandem Repeat profiling using ANSI/ATCC ASN-0002-2011 guidelines and tested Mycoplasma negative according to the MycoAlert Mycoplasma Detection Kit (Lonza). All cell lines used in this study were maintained in a 37 °C incubator, 5% CO_2_, and propagated in media containing 10% FBS and 1% NEAA in IM95—DMEM with 10 mg/L insulin, OCUM1—DMEM with 0.5 mM Na-Pyruvate, MKN1, NCC19, NCC24, NCC59, SNU484, SNU719, SNU1750, and SNU1967—RPMI.

### TCGA molecular subtype classification

Molecular subtypes of each cell lines were determined using exome data. Cell lines with > 10 EBV reads per million sequencing reads were first classified as Epstein-Barr virus positive (EBV). Next, microsatellite instability (MSI) status was determined with MSIsensor [48], with a cutoff score of 3.5 as recommended by the authors. Chromosomal instability (CIN) was inferred by the presence of whole genome doubling using ABSOLUTE [49]. The remaining cell lines were classified as genome stable (GS).

### PacBio Iso-Seq library preparation and sequencing

Total RNA was extracted from 10 gastric cell lines using RNeasy Mini Kit (QIAGEN) according to the manufacturer’s instructions. RNA was quantified by Qubit RNA BR Assay kit (Molecular Probes) and quality assessed with a 2100 Agilent Bioanalyzer using RNA Nanochip (Agilent Technologies). Only samples with RIN 8.0 and greater were selected for library preparation.

First-strand cDNA synthesis was performed using the SMARTer PCR cDNA Synthesis Kit (Clonetech Laboratories) from 4 μg of total RNA input according to the manufacturer’s instructions. A total of 12 PCR cycles of amplification was performed for each sample using PrimeSTAR GXL DNA polymerase (Clonetech laboratories). The amplified cDNA products were made into SMRTbell template libraries according to the Iso-Seq protocol by Pacific Biosciences. Sequencing was performed on the PacBio Sequel System, and 4 SMRTcells were run for each sample with a movie run-time of 600 min for each SMRTcell.

### RNA-seq library preparation and sequencing

All 10 gastric cell lines were profiled using a polyA-selected RNA sequencing (mRNA-seq) protocol. Total RNA was extracted using the Qiagen RNeasy Mini Kit (Qiagen) according to the instructions of the manufacturer. Total RNA quality check was done using the RNA 6000 LabChip Kit on the Agilent Bioanalyzer (Agilent Technologies, Palo Alto, CA). Two micrograms of total RNA was used to create libraries with Illumina TruSeq RNA Sample Prep Kit v2 (Illumina, San Diego, CA, USA) according to the manufacturer’s instructions. Samples successfully meeting the size and concentration criteria were pooled at equimolar concentrations. Two samples with unique index-tag adapter sequences were combined for multiplex NGS in each lane on the Illumina HiSeq 2000 (Illumina, San Diego, California, USA).

Twenty paired tumor-normal GC samples were profiled using a total RNA with ribosomal depletion protocol. Total RNA was extracted using the Qiagen RNeasy Mini kit. RNA-seq libraries were constructed according to the manufacturer’s instructions using Illumina Stranded Total RNA Sample Prep Kit v2 (Illumina, San Diego, CA) Ribo-Zero Gold option (Epicenter, Madison, WI) and 1 μg total RNA. Completed libraries were validated with an Agilent Bioanalyzer (Agilent Technologies, Palo Alto, CA) and applied to an Illumina flow cell via the Illumina Cluster Station. Sequencing was performed using the paired-end 101-bp read option.

### Sequence processing

For each sample, we used CCS module of IsoSeq3 program (https://github.com/PacificBiosciences/IsoSeq3) to generate circular consensus sequence (CCS) reads from the sub-reads generated from the sequencing run. Following this, the reads that were identified as full length were only considered for de novo clustering of reads using cluster module of IsoSeq3 to identify unique isoforms. These isoforms were mapped to human genome (version hg38) using GMAP [14] and only high-quality isoforms (supported by at least two FLNC reads) were considered for further analysis. Isoforms identified across all cell lines were merged into a single non-redundant transcriptome using gffcompare (https://ccb.jhu.edu/software/stringtie/gffcompare.shtml) and annotated using SQANTI2 (https://github.com/Magdoll/SQANTI2).

SQANTI2 provide annotation on CAGE peak, polyA sites, and NMD prediction. HLA binding sites were identified using netMHCpan [21], assuming a representative HLA type (HLA-A*02:01). For each coding isoform, frequency of strong binder sites is normalized to the length of its coding sequence.

Transcript-level expression in the unit of transcript per million (tpm) were estimated for each identified isoform using Kallisto [20] applied on short-read sequencing data and reference transcriptome defined using Iso-seq. For comparison between different isoform quantification strategies, tpm is also calculated using short-read sequencing data on short-read defined transcriptome and full-length reads on Iso-seq transcriptome (FL_TPM).

We use GeneMarkS-T (GMST) algorithm [50], as implemented in SQANTI2 to predict coding sequences from the generated transcript sequences. GeneMarkS-T utilizes iterative self-training and a hidden semi-Markov model to predict coding regions in eukaryotic transcripts. This algorithm had also been used in other publications analyzing human long-read transcriptomes [17, 23, 51].

### Rarefaction curve analysis

Rarefaction curves of isoform diversity were performed using the specaccum function from R library vegan. The input to specaccum is a table of Iso-seq isoforms for FSM, NIC, and NNC categories identified in the ten cell lines. For rarefaction analysis by subsampling full-length reads, relative abundances of isoforms were estimated by extracting the number of full-length sequences supporting each isoform from the FSM, NIC, and NNC categories. Rarefaction analysis was performed using the “subsample_with_category.py” script in the cDNA cupcake package.

### Alternative splicing event and alternative promoter analysis

After data processing, the full-length GC transcriptome was analyzed using the software SUPPA2 to detect 7 types of alternative splicing events including A3/A5 (alternative 3′ and 5′ splice sites), AF/AL (alternative first and last exons), SE (skipping exon), RI (retained intron), and MX (mutually exclusive exon). Specifically, SUPPA2 calculates possible alternative splicing events from an input annotation file containing the genomic coordinates and ranges of transcript isoforms (GTF format). We then used the *generateEvent* command in SUPPA2 with –f ioe options on the gtf file containing the FSM, NIC, and NNC isoforms identified from SQANTI2. This command generates an ioe output file containing the local alternative splicing events from the gtf file. Splicing events were considered as novel if all transcripts containing the splice events are novel isoforms (NIC or NNC), and splicing events found in at least one known isoform (FSM) were considered known. Expression levels of each alternative splicing event was estimated using the *psiPerEvent* command in SUPPA2, using the ioe file generated from *generateEvent* and gene expression matrix generated using Kallisto. This command generates a table containing the expression level (PSI) for each identified alternative splicing event per sample.

The full-length transcriptome was analyzed with *proActiv* version 0.1.0 (https://goekelab.github.io/proActiv/) [8] software to identify active promoter sites. Short-read RNA-seq from cell lines, primary GC, and adjacent normal samples were aligned to the reference genome using STAR [52]. The junction files and full-length transcriptome were used as input by *proActiv* to estimate promoter activities in each sample. Differentially expressed promoters were identified using DESeq2 [53]. We assembled two independent cohorts of primary GC and normal RNA-seq samples to identify differentially expressed promoters between GC and normal samples. The first cohort consists of 282 tumor and 33 normal samples from TCGA. The second cohort consists of 20 paired tumor-normal GC samples sequenced in-house.

### Mass spectrometry proteomics

Cell lines were grown and extracted in quadruplicates using RIPA buffer (Sigma) according to the manufacturer’s instructions. In total, 200 μg of protein was used for MS sample preparation. Samples were boiled at 95 °C prior to separation on a 12% NuPAGE Bis-Tris precast gel (Thermo Fisher Scientific) for 15 min at 170 V in 1× MOPS buffer. The gel was fixed using the Colloidal Blue Staining Kit (Thermo Fisher Scientific) and each lane was divided into 2 equal fractions. For in-gel digestion, samples were destained in destaining buffer (25 mM ammonium bicarbonate; 50% ethanol), reduced in 10 mM DTT for 1 h at 56 °C followed by alkylation with 55 mM iodoacetamide (Sigma) for 45 min in the dark. Tryptic digestion was performed in 50 mM ammonium bicarbonate buffer with 2 μg trypsin (Promega) at 37 °C overnight. Peptides were desalted on StageTips and analyzed by nanoflow liquid chromatography on an EASY-nLC 1200 system coupled to a Q Exactive HF mass spectrometer (Thermo Fisher Scientific). Peptides were separated on a C18-reversed phase column (25 cm long, 75 μm inner diameter) packed in-house with ReproSil-Pur C18-AQ 1.9 μm resin (Dr Maisch). The column was mounted on an Easy Flex Nano Source and temperature controlled by a column oven (Sonation) at 40 °C. A 215-min gradient from 2 to 40% acetonitrile in 0.5% formic acid at a flow of 225 nl/min was used. Spray voltage was set to 2.4 kV. The Q Exactive HF was operated with a TOP20 MS/MS spectra acquisition method per MS full scan. MS scans were conducted with 60,000 at a maximum injection time of 20 ms and MS/MS scans with 15,000 resolution at a maximum injection time of 50 ms.

The raw files were processed with MaxQuant [27], and search results were filtered with a false discovery rate of 0.01. Known contaminants, reverse hits, and entries that did not qualify as unique peptides of the MaxQuant results were removed.

### Western blotting

For *ARID1A* overexpression studies, constructs were amplified the following primers:

*ARID1A*-Canonical-F: 5′-CGACGATGACAAGGGATCCATGGCCGCGCAGGTCGCCCCCGC-3′.

*ARID1A*-Known-F-: 5′-CGACGATGACAAGGGATCCATGGGGGGAGGCGGCCCCTCCGC-3′.

ARID1A-Novel-F: 5′-CGACGATGACAAGGGATCCATGGATCAGATGGGCAAGATGAG-3′.

ARID1A-R: 5′-GGAATTGATCCCGCTCGAGTCATGACTGGCCAATCAAAAACA-3′.

PCR products were cloned into a pHR’CMVGFPIRESWSln18-based vector (gift from Dr. Shang Li) using Gibson Assembly Master Mix (NEB).

Cells were lysed in RIPA buffer (Sigma) for 10 min on ice with the presence of protease inhibitors. Cell lysates were centrifuged at 9000 rpm for 10 min and supernatants were collected for concentration measurements using Pierce BCA protein assay kit. The following antibodies were used for western blotting: ARID1A (sc-32761, Santa Cruz), GAPDH (60004-1-Ig, Proteintech Group), and TMEM59 (GTX104486, GeneTex).

### 5′ RACE

5′ Rapid amplification of cDNA ends (5′ RACE) was performed using the FirstChoice™ RNA Ligase Mediated RACE (RLM-RACE) Kit (Invitrogen, AM1700). In brief, 10 μg of total RNA was treated with calf intestinal phosphatase (CIP) to remove the 5′-phosphate from uncapped RNA (e.g., ribosomal RNA, fragmented mRNA, tRNA). Full-length, capped mRNA was unaffected in this treatment. Then the RNA is treated with tobacco acid pyrophosphatase (TAP) to remove the cap structure from the full-length mRNA, exposing its 5′-monophosphate. As the result, only the uncapped, full-length mRNAs contained a free 5′-phosphate, which was ligated with a synthetic RNA adapter. Subsequently, cDNA was synthesized from reverse transcription reaction with M-MLV Reverse Transcriptase. cDNA was then amplified by two rounds of nested PCR (namely outer and inner PCR) using adaptor-specific outer/inner primers with gene-specific outer/inner primers by Q5® High-Fidelity DNA Polymerase (NEB, M0491S). PCR amplification results were examined by gel electrophoresis. PCR bands of interest were excised and purified for cloning with the TOPO™ TA Cloning™ Kit (Invitrogen, 450640). A minimum of 7 independent colonies were selected for sequencing. Gene-specific outer/inner primers are listed in Additional File 2, Table S8.

### MeDIP-sequencing

DNA was sonicated using COVARIS S2, and peak fragment distribution between 100 and 500 bp was verified on an Agilent Bioanalyzer (Agilent Technologies) using the DNA1000 chip. Fragmented DNA was end-repaired, dA-tailed, and adapter ligated using NEBNext® DNA Library Prep Master Mix Set for Illumina (E6040). Samples were then spiked with control DNAs that were unmethylated, methylated, and hydroxymethylated (Diagenode C02040010) as a quality control measure. For each sample, input DNA that was not exposed to the primary antibody was included. Adapter-ligated DNA was subjected to immunoprecipitation with a primary monoclonal antibody against 5-methyl cytosine (Diagenode C15200081) using a previously published protocol [54]. Real-time PCR using primers against the spiked DNA controls were performed to verify successful and specific enrichment of methylated DNA (data not shown). Immunoprecipitated samples were amplified using Phusion® High-Fidelity DNA Polymerase (M0530) and NEBNext® Multiplex Oligos for Illumina® (E7335) for 10 cycles. Amplified libraries were run on the Agilent Bioanalyzer using the high-sensitivity DNA kit prior to Illumina sequencing using a single-end 100 base pair configuration.

MeDIP short reads were aligned to the reference genome using bwa [55], and duplicates were removed using samtools [56]. Peaks were called using MACS2 [57] using input control. Enrichment of MeDIP peaks around promoter sites were visualized using genomation [58].

### Clinical outcome analysis

We downloaded clinical data of GC samples from the integrated TCGA clinical data resource [59]. Then, the function “surv_cutpoint” from R package “survminer” was applied to determine the optimal promoter activity ratio based on progression-free survival information of the patients. Survival in patients with high or low promoter activity ratio was compared using the Kaplan–Meier method and the log-rank test.

### Bioinformatics workflow and command lines

The overall bioinformatics workflow is provided as Additional File 1; Figure S7 and the command lines used are listed in Additional file 3.

## Supplementary Information

**Additional file 1: Figure S1.** Rarefaction curve by sub-sampling full-length reads. **Figure S2.** Comparison between transcriptome predicted using long-read Iso-seq and short-read RNA-seq. **Figure S3.** Examples of alternative promoter usage associated with changes in the CDs, detected from mass spectrometry data. **Figure S4.** Validation of Iso-seq transcripts. **Figure S5.** Transcription factor enrichment in tumor-specific promoters. **Figure S6.** Representative meDIP-seq peak enrichment around promoter regions in IM95 and MKN1 cell lines. Figure S7. Bioinformatics workflow used in this study.

**Additional file 2: Table S1.** Molecular subtyping in gastric cancer cell lines. **Table S2.** Categories of isoform identified. **Table S3.** Correlation between predicted transcriptome (Iso-seq) with promoter activity (*proActiv*) at different gene lengths. **Table S4.** Correlation coefficient between Iso-seq and *proActiv*. **Table S5.** List of unique peptides in Iso-seq sequences and not found in Gencode v32, validated from mass spectrometer data. **Table S6.** Down-regulated promoter (tumor vs normal). **Table S7.** Up-regulated promoter (tumor vs normal). **Table S8.** Gene-specific outer/inner primers used for RLM-RACE.

**Additional file 3.** Command lines used in this study.

**Additional file 4.** Review history.

## Data Availability

Datasets generated and/or analyzed during the current study are available in SRA [PRJNA635275] [60] and GEO [GSE157750] [61]. Iso-seq, short-read RNA-seq and MeDIP-seq are included. The mass spectrometry proteomics data has been deposited into the ProteomeXchange Consortium via the PRIDE [62] partner repository under dataset identifier PXD023373 [63].
