# Supplementary material for: Long-read transcriptome sequencing reveals abundant promoter diversity in distinct molecular subtypes of gastric cancer
Source: Genome Biol. 2021 Jan 22;22:44. doi: 10.1186/s13059-021-02261-x (PMC7821541; doi:10.1186/s13059-021-02261-x)
Supplement: Supplementary file 1 — Additional file 1: Figure S1. Rarefaction curve by sub-sampling full-length reads. Figure S2. Comparison between transcriptome predicted using long-read Iso-seq and short-read RNA-seq. Figure S3. Examples of alternative promoter usage associated with changes in the CDs, detected from mass spectrometry data. Figure S4. Validation of Iso-seq transcripts. Figure S5. Transcription factor enrichment in tumor-specific promoters. Figure S6. Representative meDIP-seq peak enrichment around promoter regions in IM95 and MKN1 cell lines. Figure S7. Bioinformatics workflow used in this study. [file 13059_2021_2261_MOESM1_ESM.docx]

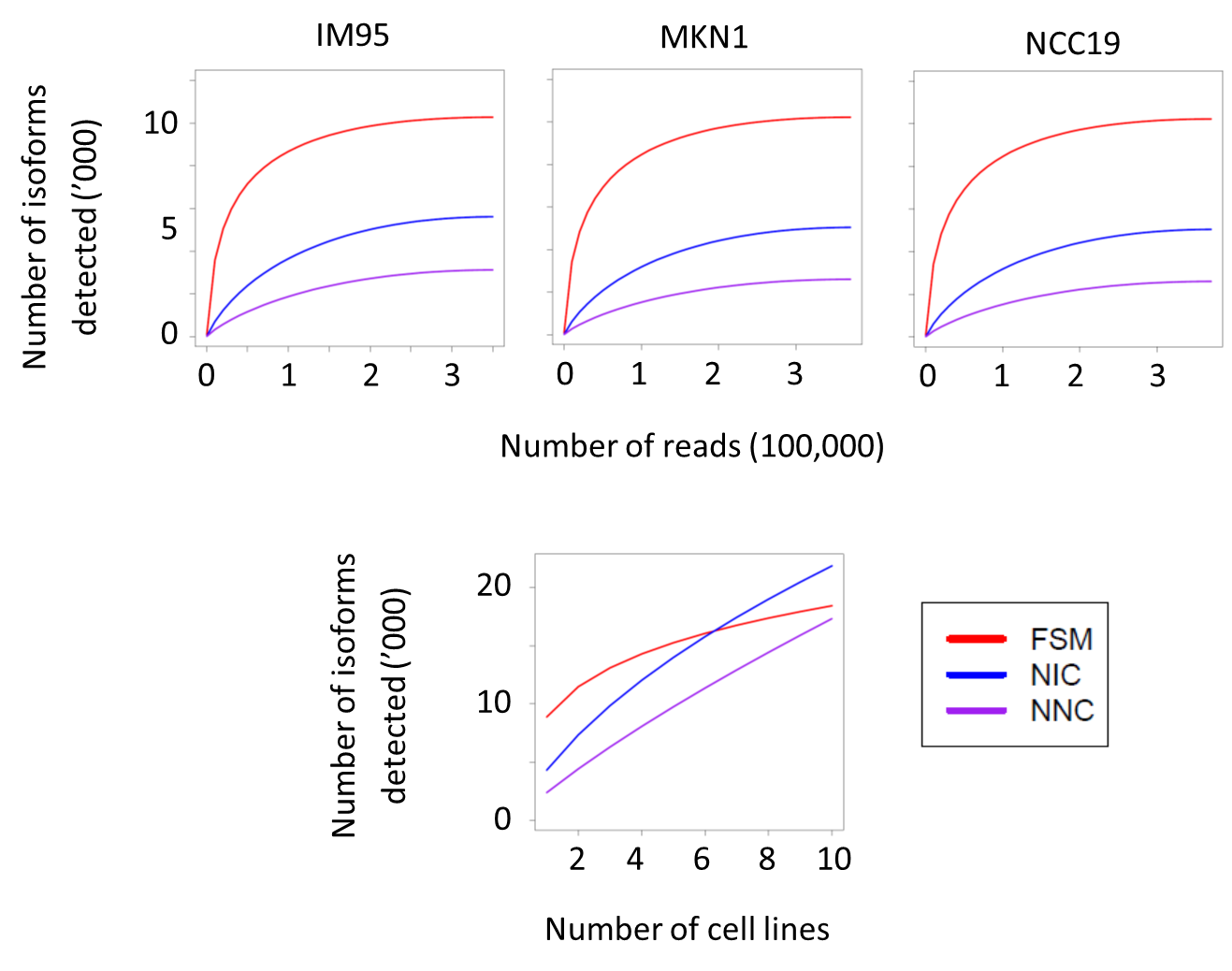


**Figure S1.** Rarefaction curve by sub-sampling full-length reads. Representative rarefaction curve by subsampling is shown in 3 cell lines (IM95, MKN1 and NCC19). Rarefaction curve from the addition of cell lines is shown at the bottom for comparison.


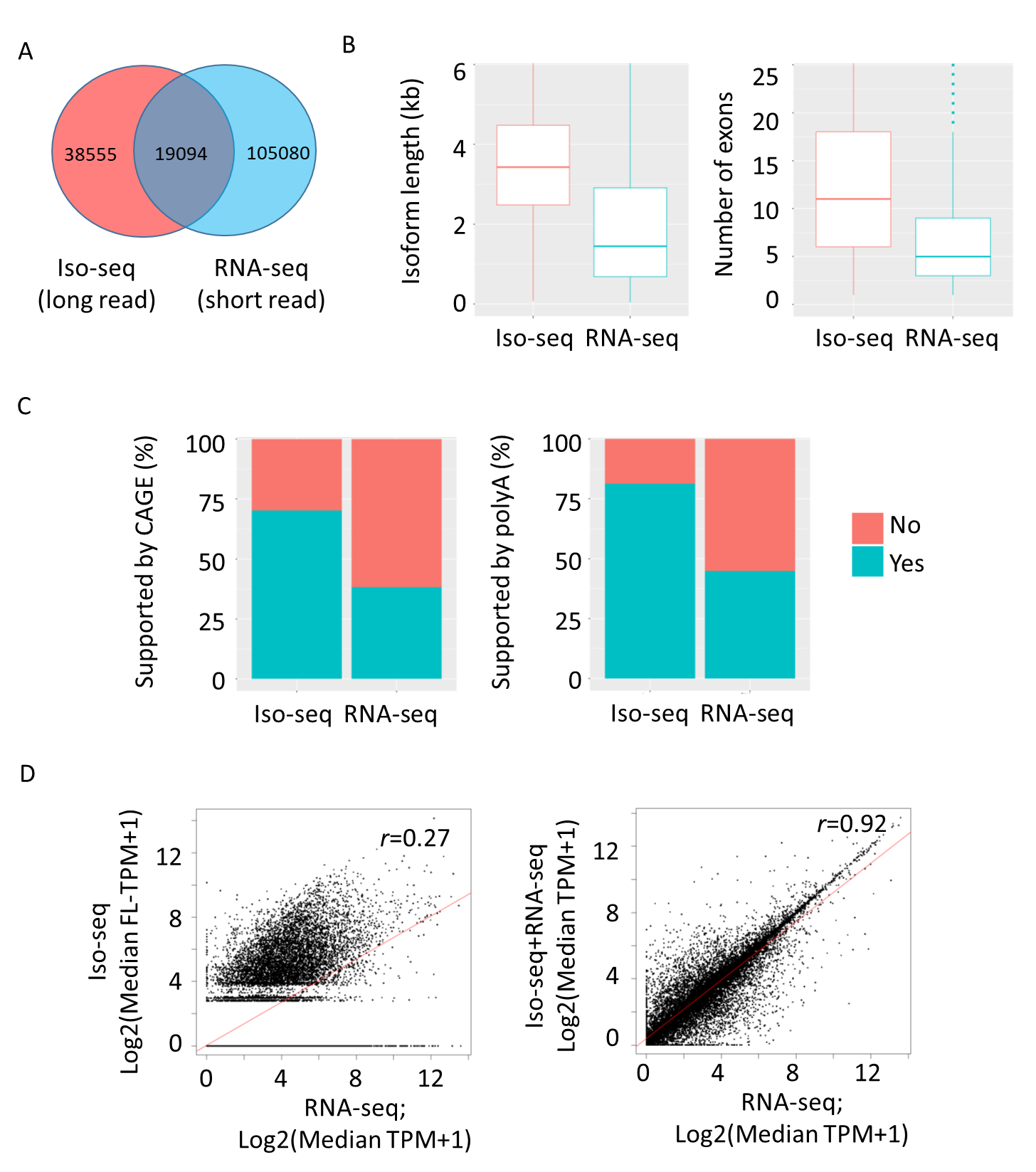


**Figure S2.** Comparison between transcriptome predicted using long-read Iso-seq and short-read RNA-seq. (a) Number of isoforms (FSM, NIC or NNC) predicted using long-read Iso-seq and short-read RNA-seq. (b) Isoform length and number of exons in Iso-seq or RNA-seq isoforms. (c) CAGE and polyA support for Iso-seq or RNA-seq isoforms. (d) Correlation between expression level estimated using Iso-seq (FL-TPM) or RNA-seq on Iso-seq transcriptome with expression level estimated with RNA-seq data alone.

**
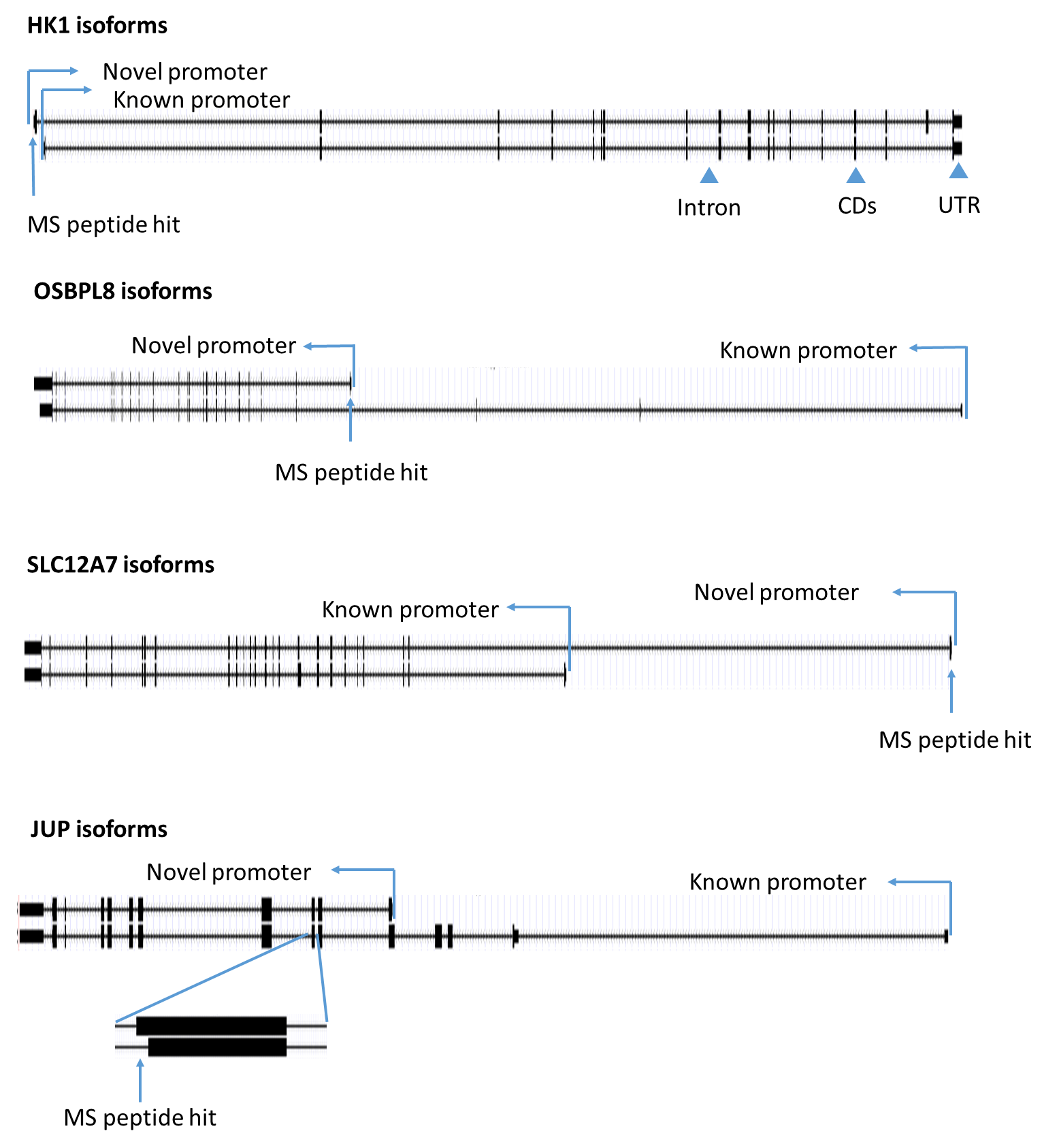
**

**Figure S3.** Examples of alternative promoter usage associated with changes in the CDs, detected from mass spectrometry data. Lines indicate introns, thin blocks indicate untranslated regions and thick blocks indicate coding exon regions. Arrows indicate isoform-specific unique peptides detected from mass spectrometry proteomics dataset.


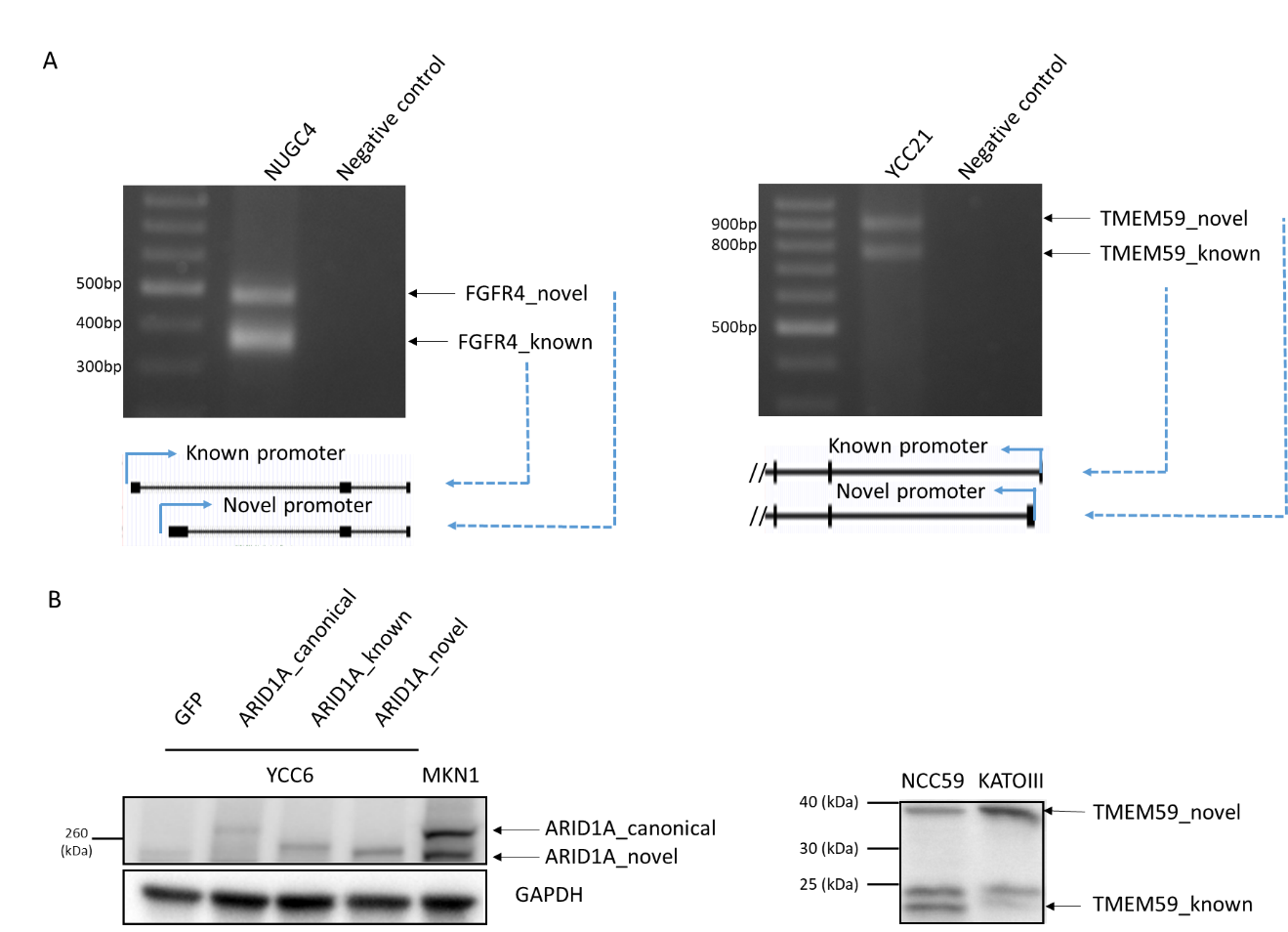
**Figure S4.** Validation of Iso-seq transcripts. (a) 5’ RACE experiments demonstrating expression of FGFR4 (left; in NUGC4 cell line) and TMEM59 (right; in YCC21 cell line) isoforms initiated from novel promoter sites. Sanger sequencing results of the 5’ RACE products are mapped to the predicted known and novel promoters. (b) Western blot demonstrating the expression of novel ARID1A (left; in MKN1 cell line; reference ARID1A isoforms were exogenously expressed in YCC6 cells to provide references) and TMEM59 proteins (right; in NCC59 and KATOIII cell lines).


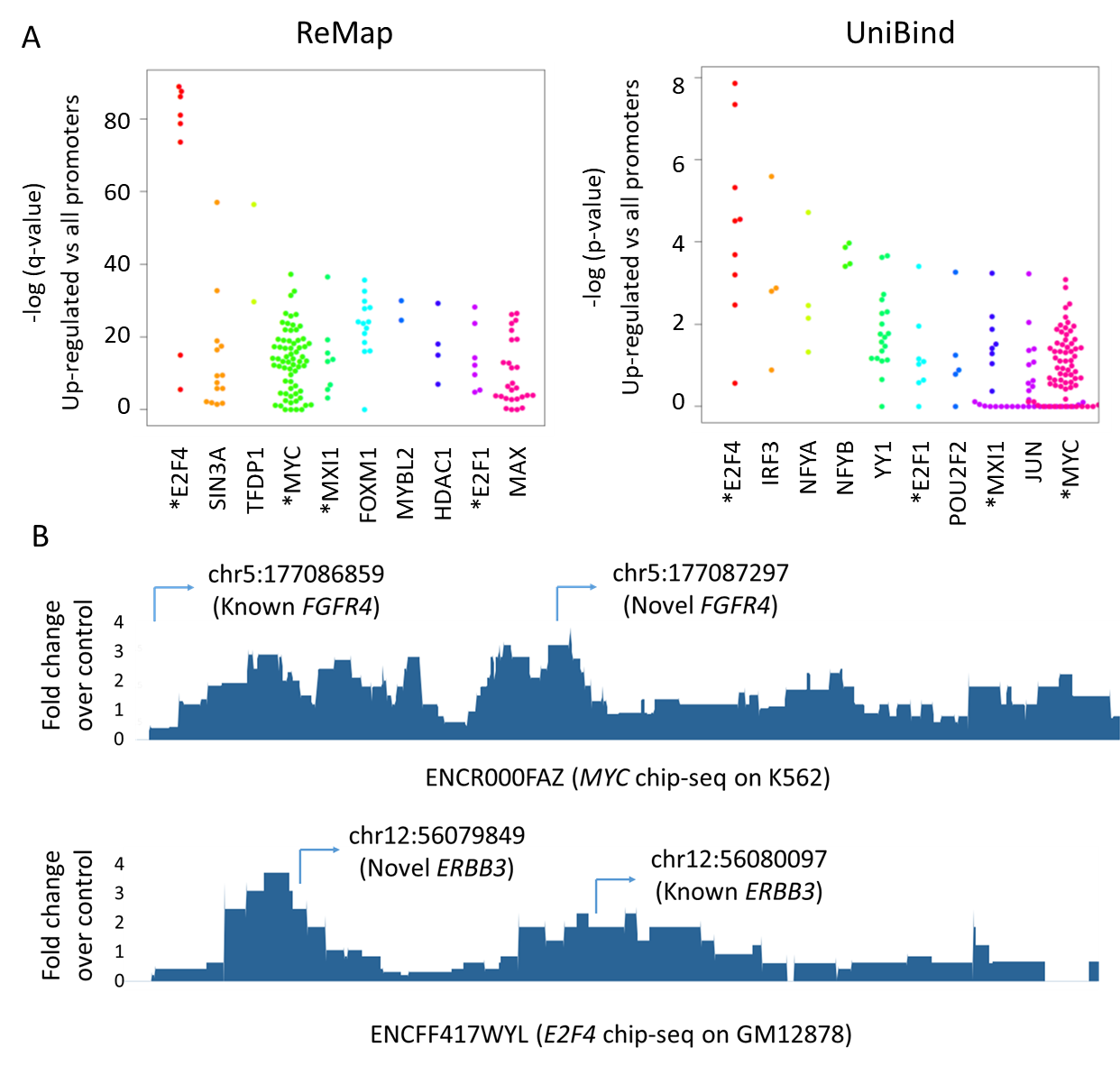


**Figure S5.** Transcription factor enrichment in tumor-specific promoters. (a) 10 highest ranked TF enrichments in up-regulated promoters compared with all promoters predicted using ReMap and UniBind. Each dot represents a ChIP-seq experiment in different cells with the labelled transcription factor. Asterisks indicate common predictions by the two methods. (b) Genome browser shows enriched MYC and E2F4 transcription factors over FGFR4 and ERBB3 promoters.


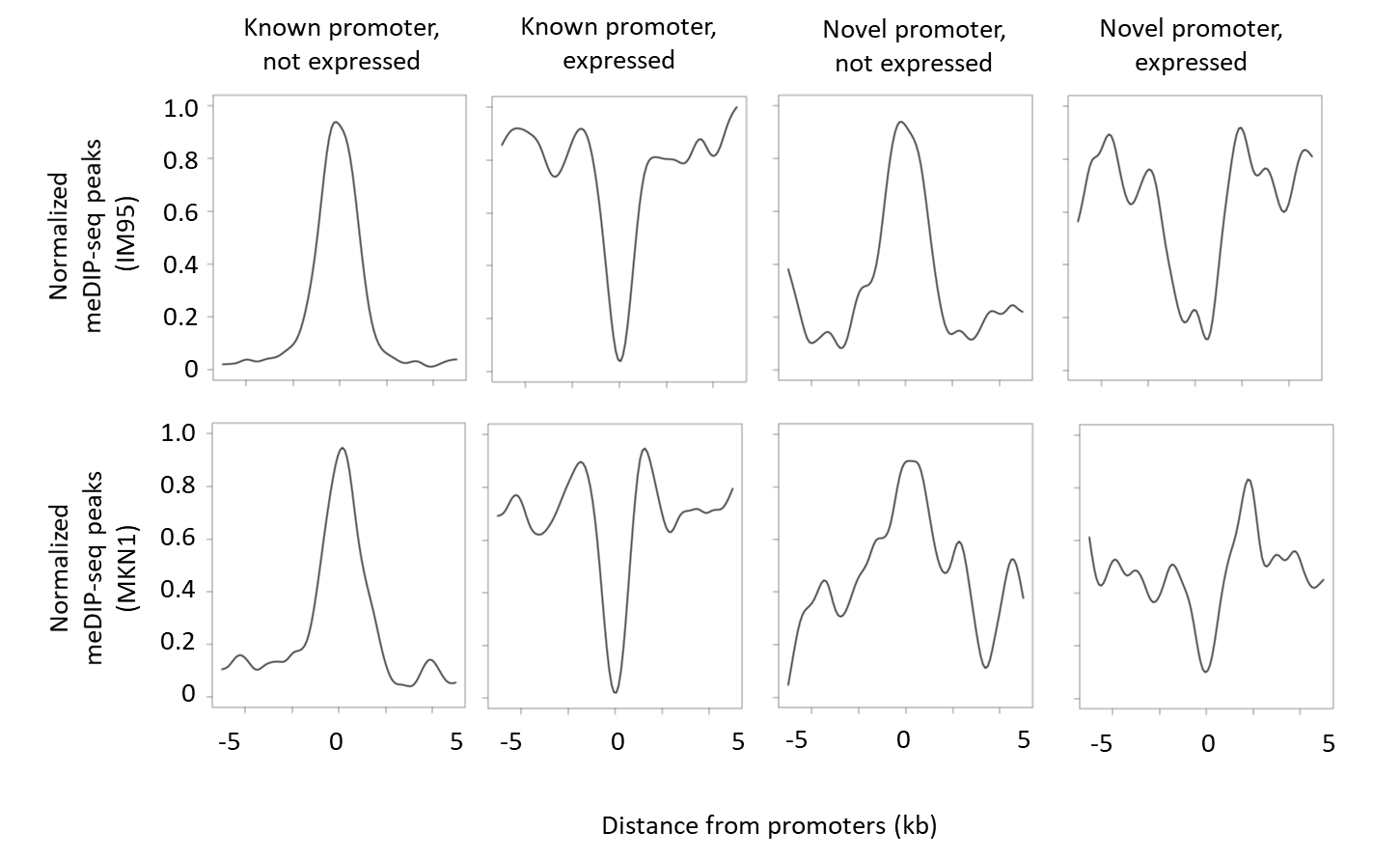


**Figure S6.** Representative meDIP-seq peak enrichment around promoter regions in IM95 and MKN1 cell lines.


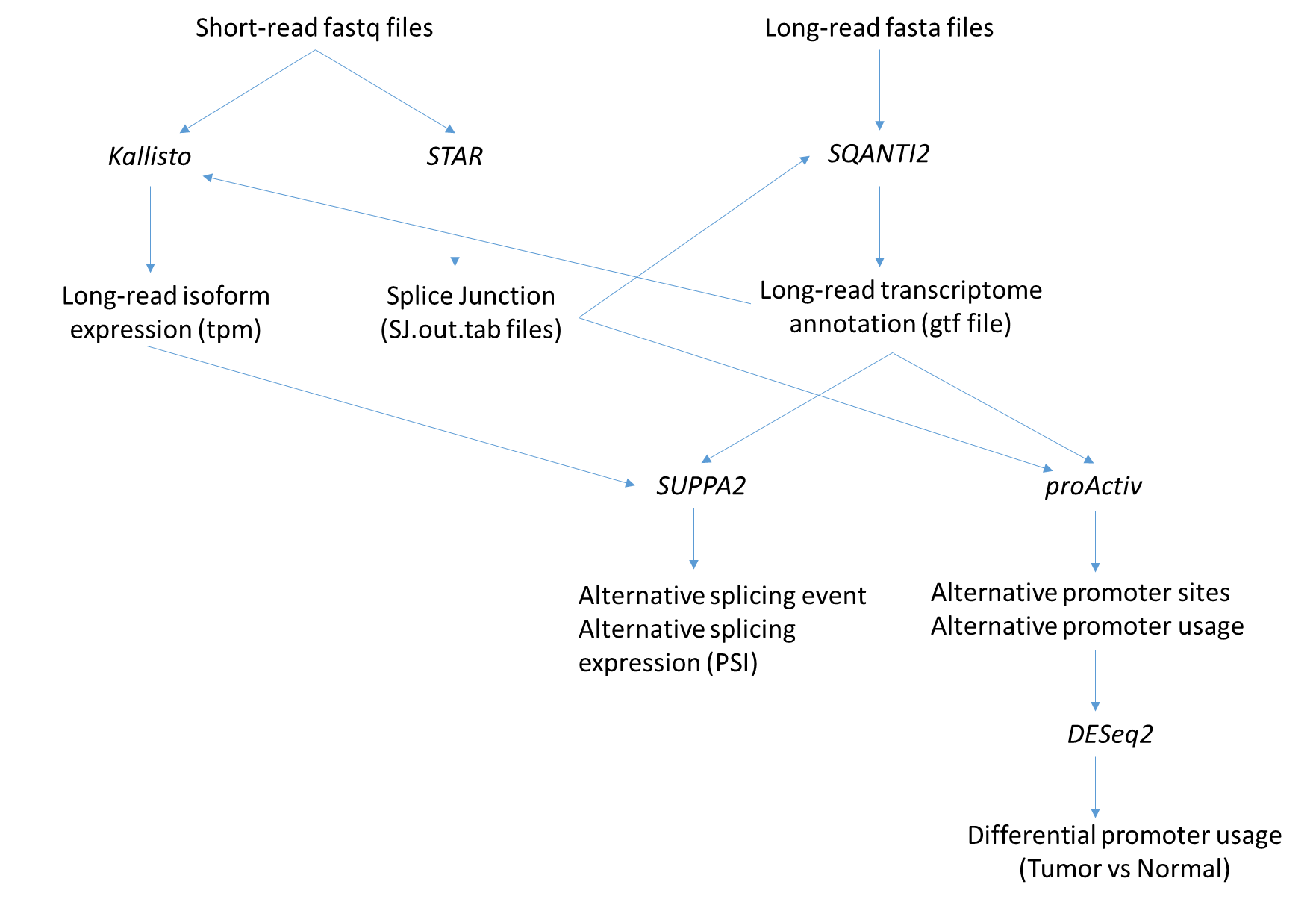


**Figure S7.** Bioinformatics workflow used in this study.
