# Supplementary material for: Long-read transcriptome sequencing reveals abundant promoter diversity in distinct molecular subtypes of gastric cancer
Source: Genome Biol. 2021 Jan 22;22:44. doi: 10.1186/s13059-021-02261-x (PMC7821541; doi:10.1186/s13059-021-02261-x)
Supplement: Supplementary file 3 — Additional file 3. Command lines used in this study. [file 13059_2021_2261_MOESM3_ESM.docx]

Command lines used in this study:

STAR

STAR \

--genomeDir [genome directory] \

--readFilesIn file1.R1.fastq.gz file1.R2.fastq.gz \

--outSAMtype BAM SortedByCoordinate \

--readFilesCommand zcat

SQANTI2

# Annotate isoforms

python sqanti_qc2.py cupcake.collapsed.filtered_corrected.fasta \

--fl_count cupcake.collapsed.filtered.abundance.txt \

gencode.v32.annotation.gtf.gz \

hg38_noALT.fa \

--coverage SJ.out.tab \

--cage_peak hg38.cage_peak_phase1and2combined_coord.bed \

--polyA_motif_list human.polyA.list.txt

# Filter isoforms

python sqanti_filter2.py \

cupcake.collapsed_classification.txt \

cupcake.collapsed.renamed_corrected.fasta \

cupcake.collapsed.renamed_corrected.sam \

cupcake.collapsed.renamed_corrected.gtf

Kallisto

kallisto index –i index [isoform fasta file]

kallisto quant -i index -o out file1.R1.fastq.gz file1.R2.fastq.gz

SUPPA2

#Identify splicing events

python suppa.py generateEvents \

–I [gtf file from SQANTI2] \

-o events \

-e SE SS MX RI FL \

-f ioe

#Calculate PSI for each splicing events

python suppa.py psiPerEvent \

-i all.events.ioe \

-e [tpm expression file from Kallisto] \

-o events

proActiv

#Identify promoter sites from gtf file

txdb <- makeTxDbFromGFF("[gtf file from SQANTI2]")

promoterAnnotationData <- preparePromoterAnnotationData(txdb, species = 'Homo_sapiens')

# The paths to SJ.out.tab files and labels for samples

starJunctionFiles <- list.files("SJ", full.names = TRUE)

starJunctionFileLabels <- c("IM95", "MKN1", …)

# Count the total number of junction reads for each promoter

promoterCounts.star <- calculatePromoterReadCounts(promoterAnnotationData,

junctionFilePaths = starJunctionFiles,

junctionFileLabels = starJunctionFileLabels,

junctionType = 'star')

# Normalize promoter read counts by DESeq2

normalizedPromoterCounts.star <- normalizePromoterReadCounts(promoterCounts.star)

# Calculate absolute promoter activity

absolutePromoterActivity.star <- getAbsolutePromoterActivity(normalizedPromoterCounts.star, promoterAnnotationData)

DESeq2

dds <- DESeqDataSetFromMatrix(countData=promoterCounts.star[activePromoters,], colData = coldata, design = ~ condition)

dds <- DESeq(dds)

res.GC.Normal <- results(dds, contrast=c("condition","Cancer","Normal"))
