# Supplementary material for: Long-read transcriptome sequencing reveals abundant promoter diversity in distinct molecular subtypes of gastric cancer
Source: Genome Biol. 2021 Jan 22;22:44. doi: 10.1186/s13059-021-02261-x (PMC7821541; doi:10.1186/s13059-021-02261-x)
Supplement: Supplementary file 4 — Additional file 4. Review history. [file 13059_2021_2261_MOESM4_ESM.docx]

Review History

**First round of review**

**Reviewer 1**

**Are you able to assess all statistics in the manuscript, including the appropriateness of statistical tests used?**

Yes, and I have assessed the statistics in my report.

**Comments to author:**

The manuscript entitled "Long-read Transcriptome Sequencing Reveals Abundant Promoter Diversity in Distinct Molecular Subtypes of Gastric Cancer" from Dr. Patrick Tan's lab analyzed alternative splicing in Gastric Cancer (GC) by performing full-length RNA-seq in 10 GC cell lines using the PacBio Iso-seq platform. Short-read RNA-seq data generated from Illumina sequencing platform were used to quantify the expression level. The authors reported novel isoforms in three different classes (i.e. novel in catalog (NIC), novel not in catalog (NNC), and incomplete-splice matches (ISM)), compared the expression level of normal versus known isoforms and analyzed the alternative promoter usage using the proActiv program that they developed previously for analyzing alternative promoter in TCGA short-read RNA-seq data. The authors then analyzed the association of promoter activity patterns to progression-free survival in the TCGA GC data set and identified a novel isoform of ARID1A to be positively correlated with poor survival.

The use of long-read sequencing technology to characterize the diversity of GC transcriptome will be of great value to the community. However, the study is largely descriptive and additional data are required to validate some of the key conclusions.

Major comments:

1) Use of Iso-seq to analyze full-length transcript in cancer cell is the main strength of the study as the analysis of alternative promoter activity largely recapitulates the results reported by the authors in a pan-cancer study of alternative promoter usage published last year (Demircioglu et al, Cell. 2019;178(6):1465-77 e17). However, the results of full-length transcript are descriptive and will require further experimental validation/data analysis in the following areas: a) alternative technology (e.g. 5'/3' RACE) to validate at least a few novel isoforms highlighted in the manuscript (e.g. ARID1A, FGFR4, MET, ERBB3). b) A comparison of Iso-seq+short-read RNA-seq versus short-read only analysis to demonstrate the value of incorporating long-read data. c) Prior study that utilizes Iso-seq to obtain full-length RNA-seq data shown detection of anti-sense RNA (Tian et al, Nat Commun. 2019 Jun 26;10(1):2789. Supplementary Figure 1). The authors need to describe how/if they remove antisense RNA in the analysis. d) Some of the tumor-only alternative splicing events may be caused by somatic alterations in the tumor genomes instead of the transcription deregulation. The authors need to perform additional analysis on the 10 GC cell lines to distinguish these two scenarios.

2) In line 219-235, the authors summarized "These results suggest that alternative promoter usage may contribute to functional diversification of the proteome by allowing for a single gene to select for multiple protein-coding sequences." This was based on the data shown in Figure 4E. It should be noted that the data projected CDS changes in a minority of isoforms (22-24%). It has been shown in previous studies that predicted protein-truncating isoforms in prominent oncogenes such as KRAS can not be validated in primary patient samples (Ma et al, Nature. 2018 Mar 15;555(7696):371-376.). Western blot validation is required for at least a subset of the novel isoforms predicted to alter the coding regions (e.g. MET, ERBB2, CD44).

3) In their 2019 pan-cancer Cell paper, the authors speculated that different transcription factor (TF) binding may have led to the use of tumor-specific alternative promoter. In the present study, the authors again reported up-regulated isoforms that utilize novel promoters of known oncogenes, such as MET, FGFR4 and ERBB3 (Fig 5c, right). As the current analysis focuses on 10 GC cell lines, it will be important for the authors to integrate publicly available ChIP-seq data (or perform ChIP-seq or ChIP-PCR) to discover and validate the TFs that led to the use of alternative promoters in these cancer cells.

4) FGFR4 N-terminus truncation in pituitary tumors described in Discussion is expected to lack most of the extracellular domain based on my review of the literatures cited by the authors. It should be noted that the truncated form of FGFR4 pituitary tumors is likely to be caused by an alternative promoter in intron 4. By contrast, FGFR4 isoforms depicted in Figure 5D appear to use an alternative promoter in intron 1 and many have identical CDS as the wild-type FGFR4. The Discussion needs to make a clear distinction between the isoform reported in pituitary tumors and the isoforms detected in GC cell lines in this study. The current presentation can be misleading in that a reader may consider the FGFR4 isoforms detected in GC have the same functional impact as those found in pituitary tumors.

5) RNA-seq quantification. The authors used the isoforms identified in Iso-seq as the transcripts of interest and run Kallisto on short-read RNA-seq data to quantify the expression level of isoforms. Is it possible that this analytical strategy may lead to loss of lowly expressed isoforms detectable only by short-read RNA-seq?

Minor comments

1) Introduction of concepts. The authors need to describe the meaning of CAGE peaks when it was first introduced in line 129. The three different classes of novel isoforms, i.e. novel in catalog (NIC), novel not in catalog (NNC), and incomplete-splice matches (ISM), need to be described in the main text at line 124 when the concepts were introduced instead of in later paragraphs (lines 147-150). A brief description on proActiv and its performance would be very helpful.

2) More details on "rarefaction curve analysis" (line 140) should be described in Methods. In Fig 1g, the y axis label of "Isoforms diversity" may need to be changed to "Number of isoforms detected" to maintain consistency with figure legend.

3) In Fig 2a, is "NNIC" a typo?

4) From Fig. 5b, the authors compared up-regulated/down-regulated with unchanged promoters. Why the percentage is different in line 243 (20.9%) and in line 244 (21.9%)?

5) Line 247, "Repeating this analysis in a second independent cohort of 20 pairs of gastric cancer samples reidentified MET and FGFR4 as up-regulated in GC samples (Fig 5c, left)". This second cohort is not described in "Methods"

6) The RNA-seq protocol did not specify whether RNA-seq was performed as mRNA-seq or total RNA with ribosomal depletion. It is important to present this information.

7) The data availability section stated that "The datasets generated and/or analyzed during the current study are available in the SRA repository [PRJNA635275]". However, PRJNA635275 does not appear to be a valid accession in SRA. As the authors stated in abstract "Our results provide a rich resource of full-length transcriptome data for deeper studies of GC and other gastrointestinal malignancies", it is important that the authors deposit the non-redundant full-length isoform data to SRA and provide the relevant link in the manuscript.

8) Line 305-306: "The known ARID1A promoter is significantly depleted in MSI subtype (log2 fold change=-0.35, p-value=3.2x10-3) perhaps reflecting the high mutation rate of ARID1A in this subtype". There could be other genes with high mutation rate in the MSI subtype. Is this a general phenomenon? If not, I suggest removing this speculation

**Reviewer 2**

**Are you able to assess all statistics in the manuscript, including the appropriateness of statistical tests used?**

No, I do not feel adequately qualified to assess the statistics.

**Comments to author:**

The manuscript by Huang et al describes the sequencing of Gastric cancer cell lines using a long read sequencing approach (Iso-seq). The authors described the transcript isoform repertoire in these cell lines, as well as novel and diverse use of promoters in the cell lines and gastric cancer in a broader sense. The long-read transcriptome resource generated in this paper is of interest, because the field of analysis of full-length transcript sequences in cancer is only beginning to emerge. However, several major issues would need to be addressed before this paper is suitable for publication in Genome Biology.

Major points:

line 108 in the paper and figure 1G. The authors sequence each cell line to around 26 Gb and go on performing an analysis of novel and known splice isoforms. A major question is whether their sequencing depth is enough to sequence the transcriptome of each individual cell line to saturation. The fact that they see new isoforms with every cell line added, may just mean that they have not sequenced each individual cell line at high enough depth. I would suggest to add the rarefaction analysis for each individual cell line by subsampling coverage (x-axis) and plot the number of isoforms for known and novel classes (FSM, NIC, NNC) on the y-axis. Such analysis would immediately make clear whether 26Gb of sequencing data per cell line is enough to reach (near) saturation in the number of isoforms. The outcome will also be of help to understand whether the many new isoforms that are found by adding data of additional cell lines is due to their diverse transcriptomes or due to a lack of coverage for the sequencing data of the individual lines.

The methods description is poor. The authors should provide a more complete description of most of their methodology. For example, the RNA isolation procedures are not listed, neither are the exact procedures for preparation of Iso-Seq libraries. The authors make use of multiple software tools to process their data (SQANTI2, SUPPA2, etc), but they do not clearly explain how these tools were used. At the very least the command lines or workflows for running these tools should be specified. It is common in genomics papers to publish a github repository or other code repository enabling interested readers to reproduce (parts) of the analysis and/or recreation of the figures and datasets described in the paper. I would suggest the authors do this and describe their methods and the rationale for using the methods carefully.

An example where the exact approach taken is unclear is given by the analysis of cancer genes, as described in lines 200 and 246. In both cases the authors list a few cancer genes that popped-up in their analysis. However, I would think that in any large scale genomic analysis known cancer genes will somehow be related to a genomics feature that is analysed (e.g. variably expressed isoforms or genes with alternative promoters, as described in this paper). There is no description of the methodology/approach that leads the authors to the identification of these genes. It is important to understand whether their finding is noteworthy and significant at all, or just one of the many insignificant results of their analysis. Please carefully describe the systematic cancer gene association analysis that (I presume) was done.

Another example of poorly described methodology/results is exemplified by the prediction of antigenic peptides resulting from the novel isoforms (line 168, results). How did the authors define the protein sequences resulting from the novel and known isoforms? Identification of the exact sequences and reading frames is not a trivial problem, but would affect the outcome of their results dramatically, if the right approach is not taken. Please describe this in detail (at least in the methods, with a referral to this in the results). Also, the authors conclude that there is increased immunogenicity of novel isoforms compared to known isoforms (line 172), while the effect seems very small (5.4 vs 5.6 MHC binding sites per kb of sequence). These results are puzzling considering that MHC binding is not necessarily evolved to only bind novel peptide sequences. Furthermore, MHC binding is not equivalent to immunogenicity, as the latter would involve a T-cell response, which is not something the authors analyzed. Please describe the experiments and results in more detail and draw careful conclusions that are put into context of key literature in the field.

The authors describe an analysis of promoter usage, as informed by their RNA sequencing experiments, and conclude that alternative promoters are frequently used in gastric cancer and may lead to expression of different proteins, possibly with different functional roles. A question on the mechanistic basis of this observation immediately arises. Have the authors taken a look at methylation of the promoter regions that are alternatively used? It would be interesting to check (e.g. based on TCGA data) whether this is correlated to alternative promoter usage.

A complex part in the manuscript concerns the analysis of splicing events across known and novel isoforms using SUPPA2. For this analysis I would think that a database of reference transcripts is needed to determine in which category a splicing event belongs. How has the analysis of splice event categories be done exactly? Is this just an outcome of the SUPPA2 tool, and if so, what does the SUPPA2 tool do exactly to determine the categorization? See also Figure 3a, the figures are clear but it is unclear how these possibilities are assigned to each transcript detected in the long-read sequencing data described in this manuscript.

In lines 214/215 the authors describe the correlation between promoter activity predicted from short-read RNA-seq with the Iso-seq predictions. It appears that the correlation are very poor and only marginally better correlation is observed if the data are from the same cell line. I would reason that these correlations do not justify the use of short-read RNA seq data to determine promoter activity, as the authors do by use of the TCGA data for gastric cancer

Minor points:

-Fig 1E: please split the bars per Number of Isoforms, so 1, 2, 3, etc, instead of grouping (2-3, 4-6)

-Fig2A, difficult to read figure, please improve resolution/quality

-line 215, unclear sentence ("....suggesting…..novel isoforms."). Do the authors mean that promoter usage can also be identified by short-read Illumina RNA seq data, because of the (poor) correlation with Iso-seq?

-Background: list of literature on long-read sequencing of transcripts appears incomplete, while this would be required to put their study in context. See here some examples of recent work describing long-read approaches for transcript sequencing in cancer. Please make sure the background section is complete and the recent literature is well described.

https://pubmed.ncbi.nlm.nih.gov/29954844/

https://pubmed.ncbi.nlm.nih.gov/30988345/

https://academic.oup.com/dnaresearch/article/26/1/55/5194314

-figure 4d: no y-axis label

-figure 6c: a label of the colors of the lines in the plots is lacking

**Authors Response**

**Point-by-point responses to the reviewers’ comments:**

Responses to Reviewers

Reviewer #1

The manuscript entitled "Long-read Transcriptome Sequencing Reveals Abundant Promoter Diversity in Distinct Molecular Subtypes of Gastric Cancer" from Dr. Patrick Tan's lab analyzed alternative splicing in Gastric Cancer (GC) by performing full-length RNA-seq in 10 GC cell lines using the PacBio Iso-seq platform. Short-read RNA-seq data generated from Illumina sequencing platform were used to quantify the expression level. The authors reported novel isoforms in three different classes (i.e. novel in catalog (NIC), novel not in catalog (NNC), and incomplete-splice matches (ISM)), compared the expression level of normal versus known isoforms and analyzed the alternative promoter usage using the proActiv program that they developed previously for analyzing alternative promoter in TCGA short-read RNA-seq data. The authors then analyzed the association of promoter activity patterns to progression-free survival in the TCGA GC data set and identified a novel isoform of ARID1A to be positively correlated with poor survival.

The use of long-read sequencing technology to characterize the diversity of GC transcriptome will be of great value to the community. However, the study is largely descriptive and additional data are required to validate some of the key conclusions.

Major comments

1) Use of Iso-seq to analyze full-length transcript in cancer cell is the main strength of the study as the analysis of alternative promoter activity largely recapitulates the results reported by the authors in a pan-cancer study of alternative promoter usage published last year (Demircioglu et al, Cell. 2019;178(6):1465-77 e17). However, the results of full-length transcript are descriptive and will require further experimental validation/data analysis in the following areas:

a) alternative technology (e.g. 5'/3' RACE) to validate at least a few novel isoforms highlighted in the manuscript (e.g. ARID1A, FGFR4, MET, ERBB3).

*Our response: We agree with the Reviewer that alternative technologies should be used to validate some of the novel isoforms. We have addressed this concern in three ways. First, to validate expression of the novel isoforms at the protein level, we have now queried an in-house mass-spectrometry proteomics dataset of the 10 cell lines analyzed by Iso-seq. Briefly, GeneMarkS-T predicted protein coding sequences for all isoforms were added to the Gencode v32 protein-coding sequence database to form a reference proteome. Unique peptides were identified using MaxQuant [1] with the use of this reference proteome. This proteomic analysis identified 930 unique peptides from 428 Iso-seq proteins that are not found in the Gencode v32 database (Additional File 1; Table S5). Importantly, we were able to validate several unique peptide sequences associated with novel promoter sites supporting the idea that many novel Iso-seq isoforms are indeed expressed at the peptide level (Additional File 1; Figure S3). Second, we have now performed RACE to validate two novel promoter isoforms (FGFR4, TMEM59; Additional File 1; Figure S4a). Third, we now also report Western blotting validation of the novel ARID1A and TMEM59 isoforms (Additional File 1; Figure S4b), and also note RACE and protein expression of the novel MET isoform has been previously reported [2]. This information has now been added to the Results (Pages 15-16, lines 335-349).*

b) A comparison of Iso-seq+short-read RNA-seq versus short-read only analysis to demonstrate the value of incorporating long-read data.

*Our response: In accordance with the Reviewer comment, we have now performed isoform discovery on Illumina short read RNA-seq data using Stringtie (reference-guided and allowing for novel isoforms) in the same 10 cell lines profiled by Iso-seq. Similar to the Iso-seq analysis, identified isoforms were annotated using SQANTI2 and grouped according to FSM, NIC and NNC categories.*

*Using gffcompare, we compared the long-read and short-read methods and found that most isoforms identified from long-reads (66.8%) cannot be readily recovered from short-read data alone (Additional File 1; Figure S2A). Moreover, although the short-read-only analysis identified larger numbers of isoforms, further examination revealed that compared to the long-read analysis, the short-read data had much shorter isoform lengths (2182 bp vs 3564 bp, t-test p-value < 2.2x10-16), contained less exons (7.2 vs 13.1, t-test p-value < 2.2x10-16), and were less strongly supported by both CAGE (38.3% vs 70.3%, Fisher test p-value < 2.2x10-16) and polyA data (45.0% vs 81.3%, Fisher test p-value < 2.2x10-16). These results suggest that many predicted short-read isoforms are incomplete fragments of full-length isoforms, rather than true isoforms (see Additional File 1; Figure S2B, C).*

*We also compared the use of Iso-seq and RNA-seq data to estimate isoform expression, focusing on the subset of isoforms identified using both methods (n=19094). We calculated isoform expression levels (tpm) using*

*1) Iso-seq data only (FL-TPM; full-length read transcript per million)*

*2) RNA-seq data using Iso-seq defined transcriptomes (TPM; kallisto)*

*3) RNA-seq data using short-read defined transcriptomes (TPM; kallisto)*

*This analysis revealed that Iso-seq derived expression level are only weakly correlated to the RNA-seq derived expression levels (Pearson r=0.27, Spearman r=0.5), which is likely due to the relatively lower depth of sequencing in Iso-seq. However, transcript expression using RNA-seq mapped reads onto Iso-seq or short-read transcriptomes were highly correlated (Pearson r=0.92, Spearman r=0.87) (see Additional File 1; Figure S2D).*

*Taken together, these analyses demonstrate that Iso-seq and RNA-seq data are highly complementary - Iso-seq allows identification of transcript isoforms that are full-length minimizing assembly errors and artefacts due incomplete fragments, while the use of high-coverage short-read RNA-seq enables a more accurate assessment of gene expression levels. This analysis has now been included in Results (Pages 10-12, lines 230-261).*

c) Prior study that utilizes Iso-seq to obtain full-length RNA-seq data shown detection of anti-sense RNA (Tian et al, Nat Commun. 2019 Jun 26;10(1):2789. Supplementary Figure 1). The authors need to describe how/if they remove antisense RNA in the analysis.

*Our response: Thank you for this comment. We agree that besides the FSM, NNC, NIC and ISM isoform categories, SQANTI2 also produce small numbers of transcripts classified as antisense (n=261; 0.4%), genic (n=304; 0.5%; isoforms that overlap with introns) and intergenic (n=316; 0.5%; isoform in intergenic regions) (see Table below; Additional File 1; Table S2 in Main Text). Previous studies have indicated that these isoforms tend to be single-exonic with higher percentages of non-canonical splice junctions, which may be caused by experimental or technical artefacts [3]. Due to these reasons and the small numbers of isoforms in these categories, our study does not consider these categories (antisense, genic, intergenic) and we have chosen to focus on alternative splicing events found in the FSM, NIC and NNC categories only. This clarification has now been included in the Results (Pages 7-8, lines 166-174).*

d) Some of the tumor-only alternative splicing events may be caused by somatic alterations in the tumor genomes instead of the transcription deregulation. The authors need to perform additional analysis on the 10 GC cell lines to distinguish these two scenarios.

*Our response: Thank you for this comment. To explore potential relationships between alternative splicing events and somatic alterations, we have now integrated the alternative splicing data with somatic mutation data (using whole exome sequencing data of the 10 lines). Somatic mutations were identified using Mutect2 on tumor-only mode, with germline subtraction using the gnomAD database and a panel of 36 normal exome samples. Somatic changes were further annotated using Funcotator, and all variants classified as Splice Site were further inspected. This analysis identified a total of 335 splice site mutations in the 10 lines. Manual inspection of these mutations in IGV highlighted 49 of these mutations which may lead to changes in splicing at the mutated exons, as detected from the Iso-seq data. This suggests that the vast majority of the splicing alterations identified (NIC and NNC isoforms; n=39,207 compared to 49) are due to transcriptional deregulation rather than somatic alterations. This information has now been added to Results (Pages 8-9, lines 189-199).*

2) In line 219-235, the authors summarized "These results suggest that alternative promoter usage may contribute to functional diversification of the proteome by allowing for a single gene to select for multiple protein-coding sequences." This was based on the data shown in Figure 4E. It should be noted that the data projected CDS changes in a minority of isoforms (22-24%). It has been shown in previous studies that predicted protein-truncating isoforms in prominent oncogenes such as KRAS can not be validated in primary patient samples (Ma et al, Nature. 2018 Mar 15;555(7696):371-376.). Western blot validation is required for at least a subset of the novel isoforms predicted to alter the coding regions (e.g. MET, ERBB2, CD44).

*Our response: Thank you for this comment. To clarify, the majority of known/novel promoter pairs do exhibit potential changes in their CDs composition (1734/2059; 84.2%) (ie number of promoter pairs). The figure of 22-24% refers to the average extent of change in CD composition between transcripts associated with known or novel promoter pairs. This has now been clarified in the Results (Page 15, lines 328-333).*

*As described in Comment #1, we agree with the Reviewer that alternative technologies should be used to validate some of the novel isoforms. We have addressed this concern in three ways. First, to validate expression of the novel isoforms at the protein level, we have now queried an in-house mass-spectrometry proteomics dataset of the 10 cell lines analyzed by Iso-seq. Briefly, GeneMarkS-T predicted protein coding sequences for all isoforms were added to the Gencode v32 protein-coding sequence database to form a reference proteome. Unique peptides were identified using MaxQuant [1] with the use of this reference proteome. This proteomic analysis identified 930 unique peptides from 428 Iso-seq proteins that are not found in the Gencode v32 database (Additional File 1; Table S5). Importantly, we were able to validate several unique peptide sequences associated with novel promoter sites (Additional File 1; Figure S3), supporting the idea that a subset of the novel Iso-seq isoforms are indeed expressed at the peptide level. Second, we have now performed RACE to validate two novel promoter isoforms (FGFR4, TMEM59). Third, we also report Western blotting validation of the novel ARID1A and TMEM59 isoforms, and also note RACE and protein expression of the novel MET isoform has been previously reported [2]. This information has now been added to the Results (Pages 15-16, lines 335-349).*

3) In their 2019 pan-cancer Cell paper, the authors speculated that different transcription factor (TF) binding may have led to the use of tumor-specific alternative promoter. In the present study, the authors again reported up-regulated isoforms that utilize novel promoters of known oncogenes, such as MET, FGFR4 and ERBB3 (Fig 5c, right). As the current analysis focuses on 10 GC cell lines, it will be important for the authors to integrate publicly available ChIP-seq data (or perform ChIP-seq or ChIP-PCR) to discover and validate the TFs that led to the use of alternative promoters in these cancer cells.

*Our response: We agree that this is a very interesting analysis. In accordance with the Reviewer’s comment, we have now queried ReMap [4] to identify TFs enriched at tumor specific alternative promoters. The ReMap 2018 atlas contains 485 transcriptional regulator genomic occupancy profiles, derived from curated ChIP-seq, ChIP-exo DAP-seq, and ENCODE databases across 346 human cell types from 2829 ChIP-seq datasets. We integrated the 485 TF occupancy profiles against significantly up-regulated alternative promoters (FDR<0.001; n=2389) compared to all promoters identified in this study (n=18293) using the ReMapEnrich R package (https://github.com/remap-cisreg/ReMapEnrich). From 485 TFs available in Remap, 204 TFs were found to be significantly increased (q<0.001) at up-regulated promoters in at least one ChIP experiment. To assess the robustness of our analysis, we also performed the same analysis using a different TF-DNA direct interaction database (UniBind). UniBind contains information on 231 TFs from 1983 ChIP-seq datasets. Four of the 10 highest-ranked TFs by ReMap were also predicted by the UniBind_enrichment tool (https://unibind.uio.no/enrichment/), including E2F4, E2F1, MYC and MXI1 (Figure S5a, S5b). These may highlight possible TFs regulating the use of alternative promoters in gastric cancer, and these results have been included in Results (Pages 17-18, lines 387-402).*

4) FGFR4 N-terminus truncation in pituitary tumors described in Discussion is expected to lack most of the extracellular domain based on my review of the literatures cited by the authors. It should be noted that the truncated form of FGFR4 pituitary tumors is likely to be caused by an alternative promoter in intron 4. By contrast, FGFR4 isoforms depicted in Figure 5D appear to use an alternative promoter in intron 1 and many have identical CDS as the wild-type FGFR4. The Discussion needs to make a clear distinction between the isoform reported in pituitary tumors and the isoforms detected in GC cell lines in this study. The current presentation can be misleading in that a reader may consider the FGFR4 isoforms detected in GC have the same functional impact as those found in pituitary tumors.

*Our response: We apologize for this confusion. In accordance with the Reviewer’s comment, we have now clarified in the manuscript that the MET and FGFR4 isoforms described in the Discussion are *not* the same isoforms found in the Results section of this study. Specifically, we have amended the text as follows: “Notably, activation of N-terminal truncated isoforms for MET and FGFR4 have been previously reported before in human musculoskeletal [5] and pituitary tumors [6]. These isoforms have distinct transcription start sites from the MET and FGFR4 isoforms reported in this study.” (Page 23, lines 519-520)*

5) RNA-seq quantification. The authors used the isoforms identified in Iso-seq as the transcripts of interest and run Kallisto on short-read RNA-seq data to quantify the expression level of isoforms. Is it possible that this analytical strategy may lead to loss of lowly expressed isoforms detectable only by short-read RNA-seq?

*Our response: We thank the Reviewer for the comment. We acknowledge that the use of Iso-seq may have led to the omission of lowly expressed isoforms due to the lower depth of Iso-seq methods - this limitation is now reflected in the Discussion. However, as we show in Comment #1, short-read sequencing data when analysed alone also does not reliably identify full-length transcript sequences, limiting their utility for isoform discovery. Thus, the use of Iso-seq and other long-read technologies, despite their reduced sensitivities, will facilitate full length transcript identification which is critical to characterize complex alternative splicing events.*

*“Our study has several limitations. First, the sequencing depth from Iso-seq method is not yet sufficient to cover the full scope of isoform diversity. Thus, our analysis may have missed genes or isoforms expressed at low levels. Second, owing to their relatively lower sequencing coverage and potential gene length biases, long-read sequencing are less reliable in determining expression levels than short-read RNA-seq. This may explain the lower than expected correlation of promoter usage from the two methods. Despite these limitations, our results show that Iso-seq methods provide useful information that complement conventional short-read RNA-seq methods.” (Page 25, lines 543-550)*

Minor comments

1) Introduction of concepts. The authors need to describe the meaning of CAGE peaks when it was first introduced in line 129.

*Our response: Thank you for this comment. We have now revised the Results to describe CAGE in line 129. “We used various quality features provided by SQANTI2 to assess the reliability of the full-length isoforms, including non-canonical junction usage, intrinsic sequencing properties (i.e. number of predicted reverse transcriptase template switching artifacts), functional genomic evidence (i.e. overlap of 5’ transcript ends with independently published Cap Analysis of Gene Expression (CAGE) data [7] (CAGE comprises tag sequencing data directly measuring the 5’ end of transcripts), and 3’ ends with polyA tails (Fig 1d).” (Page 7, lines 158-159)*

The three different classes of novel isoforms, i.e. novel in catalog (NIC), novel not in catalog (NNC), and incomplete-splice matches (ISM), need to be described in the main text at line 124 when the concepts were introduced instead of in later paragraphs (lines 147-150).

*Our response: Thank you for this comment. We have now moved the description of the three isoform classes to line 124 from lines 147-150, as follows: “Among the isoforms, 31% (18442) were full-splice matches (FSM) matching perfectly to known transcripts, and 37% (21874), 29% (17333), and 3% (1709) were novel in catalog (NIC); corresponding to isoforms with at least one unannotated splice site, novel not in catalog (NNC); corresponding to isoforms with known splice sites but novel splice junctions, and incomplete-splice matches (ISM); corresponding to isoforms that match to a subsection of a known transcript (Fig 1c).” (Pages 6-7, lines 148-154)*

*…*

*“We proceeded to characterize the novel transcript isoforms. Of 39,207 novel isoforms, 17,333 (44%) were classified as NNC and the remaining novel isoforms were NIC.”*

A brief description on proActiv and its performance would be very helpful.

*Our response: Thank you for this comment. We have now added a brief description on proActiv and its performance, as follows: “Given the enrichment of alternate promoters in novel isoforms and their high inter-sample variability, we focused in-depth on this specific splicing sub-class. Here, we applied proActiv [8], an R package that estimates promoter activity from aligned RNA-seq data applied onto a reference transcriptome. Briefly, proActiv quantifies promoter expression using a set of unique junction reads, and we have previously shown that promoter activity predicted by proActiv shows higher consistency with CAGE and H3K4me3 histone data when benchmarked to other methods.” (Page 13, lines 294-300)*

2) More details on "rarefaction curve analysis" (line 140) should be described in Methods. In Fig 1g, the y axis label of "Isoforms diversity" may need to be changed to "Number of isoforms detected" to maintain consistency with figure legend.

*Our response: Thank you for this comment. We have now added a description of the rarefaction analysis in the Methods, and the label “Isoforms diversity” had been amended to “Number of isoforms detected” as suggested.*

*Rarefaction curve analysis*

*Rarefaction curves of isoform diversity were performed using the specaccum function from R library vegan. The input to speccacum is a table of Iso-seq isoforms for FSM, NIC and NNC categories identified in the ten cell lines. For rarefaction analysis by subsampling full-length reads, relative abundances of isoforms were estimated by extracting the number of full-length sequences supporting each isoform from the FSM, NIC and NNC categories. Rarefaction analysis was performed using the “subsample_with_category.py” script in the cDNA cupcake package. (Methods, Pages 30-31, lines 648-655)*

3) In Fig 2a, is "NNIC" a typo?

*Our response: We thank the reviewer for highlighting this typo, and we have corrected “NNIC” to “NNC”.*

4) From Fig. 5b, the authors compared up-regulated/down-regulated with unchanged promoters. Why the percentage is different in line 243 (20.9%) and in line 244 (21.9%)?

*Our response: We thank the Reviewer for highlighting this typo. We have corrected the 21.9% in line 244 to 20.9%.*

5) Line 247, "Repeating this analysis in a second independent cohort of 20 pairs of gastric cancer samples reidentified MET and FGFR4 as up-regulated in GC samples (Fig 5c, left)". This second cohort is not described in "Methods"

*Our response: We thank the Reviewer for this comment. We have now added a description of the cohorts to the Methods, as follows: “We assembled two independent cohorts of primary GC and normal RNA-seq samples to identify differentially expressed promoters between GC and normal samples. The first cohort consists of 282 tumor and 33 normal samples from TCGA. The second cohort consists of 20 paired tumor-normal GC samples sequenced in-house.” (Page 32, lines 679-682)*

6) The RNA-seq protocol did not specify whether RNA-seq was performed as mRNA-seq or total RNA with ribosomal depletion. It is important to present this information.

*Our response: In accordance with the Reviewer’s comment we have now added this information to the Methods section in the RNA-seq protocol. Specifically,*

*“All 10 gastric cell lines were profiled using a polyA-selected RNA sequencing (mRNA-seq) protocol. Total RNA was extracted using the Qiagen RNeasy Mini Kit (Qiagen) according to the instructions of the manufacturer. Total RNA quality check was done using the RNA 6000 LabChip Kit on the Agilent Bioanalyzer (Agilent Technologies, Palo Alto, CA). Two micrograms of total RNA was used to create libraries with Illumina TruSeq RNA Sample Prep Kit v2 (Illumina, San Diego, California, USA) according to manufacturer’s instructions. Samples successfully meeting the size and concentration criteria were pooled at equimolar concentrations. Two samples with unique index-tag adapter sequences were combined for multiplex NGS in each lane on the Illumina HiSeq 2000 (Illumina, San Diego, California, USA).*

*20 paired tumor-normal GC samples were profiled using a total RNA with ribosomal depletion protocol. Total RNA was extracted using the Qiagen RNeasy Mini kit. RNA-seq libraries were constructed according to manufacturer’s instructions using Illumina Stranded Total RNA Sample Prep Kit v2 (Illumina, San Diego, CA) Ribo-Zero Gold option (Epicentre, Madison, WI) and 1 μg total RNA. Completed libraries were validated with an Agilent Bioanalyzer (Agilent Technologies, Palo Alto, CA) and applied to an Illumina flow cell via the Illumina Cluster Station. Sequencing was performed using the paired-end 101 bp read option.” (Pages 28-29, lines 600-618)*

7) The data availability section stated that "The datasets generated and/or analyzed during the current study are available in the SRA repository [PRJNA635275]". However, PRJNA635275 does not appear to be a valid accession in SRA. As the authors stated in abstract "Our results provide a rich resource of full-length transcriptome data for deeper studies of GC and other gastrointestinal malignancies", it is important that the authors deposit the non-redundant full-length isoform data to SRA and provide the relevant link in the manuscript.

*Our response: We thank the Reviewer for this comment and apologize for the confusion. While this manuscript is in peer-review, the data sets are not publicly available but are accessible to reviewers, as per standard practise using the following Reviewer link (https://dataview.ncbi.nlm.nih.gov/object/PRJNA635275?reviewer=ajjavfdnfhucmct271391p5ooc). The non-redundant full-length isoform data can be accessed from the linked GEO entry https://www.ncbi.nlm.nih.gov/geo/query/acc.cgi?acc=GSE157750 using the secure token gtwjkeqklbarjij. This information had been added to the main text (Availability of data and materials, page 37, line 765).*

8) Line 305-306: "The known ARID1A promoter is significantly depleted in MSI subtype (log2 fold change=-0.35, p-value=3.2x10-3) perhaps reflecting the high mutation rate of ARID1A in this subtype". There could be other genes with high mutation rate in the MSI subtype. Is this a general phenomenon? If not, I suggest removing this speculation

*Our response: We thank the Reviewer for this guidance and have deleted the speculation, amending the relevant sentence to “The known ARID1A promoter is significantly depleted in the MSI subtype (log2 fold change=-0.35, p-value=3.2x10-3).”*

Reviewer #2

The manuscript by Huang et al describes the sequencing of Gastric cancer cell lines using a long read sequencing approach (Iso-seq). The authors described the transcript isoform repertoire in these cell lines, as well as novel and diverse use of promoters in the cell lines and gastric cancer in a broader sense. The long-read transcriptome resource generated in this paper is of interest, because the field of analysis of full-length transcript sequences in cancer is only beginning to emerge. However, several major issues would need to be addressed before this paper is suitable for publication in Genome Biology.

Major points:

line 108 in the paper and figure 1G. The authors sequence each cell line to around 26 Gb and go on performing an analysis of novel and known splice isoforms. A major question is whether their sequencing depth is enough to sequence the transcriptome of each individual cell line to saturation. The fact that they see new isoforms with every cell line added, may just mean that they have not sequenced each individual cell line at high enough depth. I would suggest to add the rarefaction analysis for each individual cell line by subsampling coverage (x-axis) and plot the number of isoforms for known and novel classes (FSM, NIC, NNC) on the y-axis. Such analysis would immediately make clear whether 26Gb of sequencing data per cell line is enough to reach (near) saturation in the number of isoforms. The outcome will also be of help to understand whether the many new isoforms that are found by adding data of additional cell lines is due to their diverse transcriptomes or due to a lack of coverage for the sequencing data of the individual lines.

*Our response: We thank the Reviewer for raising this point. In accordance with the Reviewer’s suggestion, we have now performed a rarefaction analysis by subsampling each individual cell line. Relative abundances of isoforms were estimated by extracting the number of full-length sequences supporting each isoform from the FSM, NIC and NNC categories. Rarefaction analysis was performed using the “subsample_with_category.py” script in the cDNA cupcake package. As shown in Additional File 1; Figure S1, we found that for each cell line, at the sequencing depth of 26 Gb the discovery of these isoforms reaches saturation. Thus, the increase in novel isoforms across cell lines is more likely attributable to cell-line specific transcriptomes rather than a lack of coverage in the individual cell lines. This information is now presented in Results (Page 8, lines 180-186).*

The methods description is poor. The authors should provide a more complete description of most of their methodology. For example, the RNA isolation procedures are not listed, neither are the exact procedures for preparation of Iso-Seq libraries. The authors make use of multiple software tools to process their data (SQANTI2, SUPPA2, etc), but they do not clearly explain how these tools were used. At the very least the command lines or workflows for running these tools should be specified. It is common in genomics papers to publish a github repository or other code repository enabling interested readers to reproduce (parts) of the analysis and/or recreation of the figures and datasets described in the paper. I would suggest the authors do this and describe their methods and the rationale for using the methods carefully.

*Our response: We agree with this Reviewer comment. In accordance with the Reviewer’s suggestion, we have now provided more details on the RNA isolation and preparation. A workflow of the software tools is also now provided (Additional File 1; Figure S7) along with the command lines used (Additional File 2).*

*PacBio Iso-Seq library preparation and sequencing*

*Total RNA was extracted from 10 gastric cell-lines using RNeasy Mini Kit (QIAGEN) according to manufacturer’s instructions. RNA was quantified by Qubit RNA BR Assay kit (Molecular Probes) and quality assessed with a 2100 Agilent Bioanalyser using RNA Nanochip (Agilent Technologies). Only samples with RIN 8.0 and greater were selected for library preparation.*

*First strand cDNA synthesis was performed using the SMARTer PCR cDNA Synthesis Kit (Clonetech Laboratories) from 4ug of total RNA input according to manufacturer’s instructions. A total of 12 PCR cycles of amplification was performed for each sample using PrimeSTAR GXL DNA polymerase (Clonetech laboratories). The amplified cDNA products were made into SMRTbell template libraries according to the Iso-Seq protocol by Pacific Biosciences. Sequencing was performed on the PacBio Sequel System and 4 SMRTcells were run for each sample with a movie run-time of 600min for each SMRTcell.*

*RNA-seq library preparation and sequencing*

*All 10 gastric cell lines were profiled using a polyA-selected RNA sequencing (mRNA-seq) protocol. Total RNA was extracted using the Qiagen RNeasy Mini Kit (Qiagen) according to the instructions of the manufacturer. Total RNA quality check was done using the RNA 6000 LabChip Kit on the Agilent Bioanalyzer (Agilent Technologies, Palo Alto, CA). Two micrograms of total RNA was used to create libraries with illumina TruSeq RNA Sample Prep Kit v2 (illumina, San Diego, California, USA) according to manufacturer’s instructions. Samples successfully meeting the size and concentration criteria were pooled at equimolar concentrations. Two samples with unique index-tag adapter sequences were combined for multiplex NGS in each lane on the illumina HiSeq 2000 (Illumina, San Diego, California, USA).*

*20 paired tumor-normal GC samples were profiled using a total RNA with ribosomal depletion protocol. Total RNA was extracted using the Qiagen RNeasy Mini kit. RNA-seq libraries were constructed according to manufacturer’s instructions using Illumina Stranded Total RNA Sample Prep Kit v2 (Illumina, San Diego, CA) Ribo-Zero Gold option (Epicentre, Madison, WI) and 1 μg total RNA. Completed libraries were validated with an Agilent Bioanalyzer (Agilent Technologies, Palo Alto, CA) and applied to an Illumina flow cell via the Illumina Cluster Station. Sequencing was performed using the paired-end 101 bp read option.*

*This information is now presented in Methods, Pages 28-29, lines 585-618.*

An example where the exact approach taken is unclear is given by the analysis of cancer genes, as described in lines 200 and 246. In both cases the authors list a few cancer genes that popped-up in their analysis. However, I would think that in any large scale genomic analysis known cancer genes will somehow be related to a genomics feature that is analysed (e.g. variably expressed isoforms or genes with alternative promoters, as described in this paper). There is no description of the methodology/approach that leads the authors to the identification of these genes. It is important to understand whether their finding is noteworthy and significant at all, or just one of the many insignificant results of their analysis. Please carefully describe the systematic cancer gene association analysis that (I presume) was done.

*Our response: We agree with this Reviewer comment and apologize for the lack of detail. We have amended the text to describe the specific approach we used that led to the identification of the genes in lines 200 and 246, as follows:*

*“We also used SUPPA2 to calculate relative expression levels (represented by “percent-spliced in”; PSI) and variation in expression among the different splicing classes. Across the GC lines, novel splice events of every splicing class exhibited lower expression levels compared to known splice events (average PSI 0.38 vs 0.69 in known isoforms; paired t-test p-value 6.4x10-4). However, among the splicing classes, novel AF, AL and MX (average PSI 0.46-0.47) events exhibited relatively higher expression than other splicing event types (average PSI 0.31-0.37; t-test p-value 1.5x10-3) (Fig 3c). Novel isoforms tended to be expressed more variably (average standard deviation 0.15 vs 0.12; paired t-test p-value 1.1x10-3), with AF, AL, and MX events showing the highest variance across lines (average standard deviation 0.20 vs 0.11; t-test p-value 0.011) (Fig 3d). Hierarchical clustering across the lines using the top 50 most variable splice events, as determined by standard deviation across the ten cell lines revealed that the most highly variable isoforms are often novel isoforms associated with AF events. These findings were robust even when the top 500-1000 most variable splicing events were analysed (data not shown). Gene ontology analysis of isoforms exhibiting the top 1000 splice events with the highest variance revealed that these isoforms are enriched for pathways known to be deregulated in GC, such as developmental processes and cell adhesion (gene length bias-adjusted p-values 4.9x10-8 and 8.6x10-6) (Fig 3e), including several known cancer genes such as MADD, PTK2 and NUMA1 (Fig 3f). (Pages 12-13, lines 274-291)*

*Using proActiv, we then extended our full-length transcript-informed promoter predictions to the TCGA GC RNAseq dataset (282 gastric cancer and 33 normal samples). We observed that promoter activity is distinct between tumor and normal samples, and also between different molecular subtypes of GC (Fig 5a). We then applied DESeq2 to perform differential promoter usage analysis on the tumor and normal samples. Comparison between tumor and normal samples revealed 2389 up-regulated and 2049 down-regulated isoforms in GC (FDR< 1x10-3, Additional file 1, Tables S5 and S6). Notably, promoters up-regulated in GC (n=2389) were significantly more likely to have changes in their CDs (average extent of altered CDs per promoter pair, 27.5% vs 20.9%; t-test p-value 3.3x10-8) and associated with cancer-related gene ontologies such as chromosomal organization (gene-length-adjusted p-value 5.4x10-38) and cell cycle (gene-length-adjusted p-value 3.2x10-43). We observed up-regulated isoforms comprising novel promoter isoforms of known oncogenes, such as MET, FGFR4 and ERBB3 (Fig 5c, right). Repeating this analysis in a second independent cohort of 20 pairs of gastric cancer samples re-identified MET and FGFR4 as up-regulated in GC samples (Fig 5c, left). The novel MET variant identified here has also been reported elsewhere [2, 9, 10], further validating the ability of our pipeline to identify novel cancer-associated promoters. Down-regulated promoters were not associated with CDs changes (21.8% vs 20.9%; t-test p-value 0.42) (Fig 5d). (Page 16-17, lines 353-369).*

Another example of poorly described methodology/results is exemplified by the prediction of antigenic peptides resulting from the novel isoforms (line 168, results). How did the authors define the protein sequences resulting from the novel and known isoforms? Identification of the exact sequences and reading frames is not a trivial problem, but would affect the outcome of their results dramatically, if the right approach is not taken. Please describe this in detail (at least in the methods, with a referral to this in the results).

*Our response: We agree with this Reviewer comment, and have now described the approach to define protein sequences resulting from the novel and known isoforms. Importantly, this same approach has been used in other long-read transcriptome papers, and the relevant references have been cited. The amended text now reads: “We used GeneMarkS-T (GMST) algorithm [11], as implemented in SQANTI2 to predict coding sequences from the generated transcript sequences. GeneMarkS-T utilizes iterative self-training and a hidden semi-Markov model to predict coding regions in eukaryotic transcripts. This algorithm had also been used in other publications analyzing human long-read transcriptomes [3, 12, 13].” (Methods, Page 30, lines 642-646)*

Also, the authors conclude that there is increased immunogenicity of novel isoforms compared to known isoforms (line 172), while the effect seems very small (5.4 vs 5.6 MHC binding sites per kb of sequence). These results are puzzling considering that MHC binding is not necessarily evolved to only bind novel peptide sequences. Furthermore, MHC binding is not equivalent to immunogenicity, as the latter would involve a T-cell response, which is not something the authors analyzed. Please describe the experiments and results in more detail and draw careful conclusions that are put into context of key literature in the field.

*Our response: We agree with the Reviewer that this result is still quite preliminary. We have thus toned down our phrasing of the Results and are also open to deleting the MHC results from the manuscript, subject to the Reviewer’s guidance. The amended text now reads: “Novel isoforms were also expressed at lower levels compared to known isoforms (median TPM 0.54 vs 3.08; Wilcoxon-test, p-value < 2.2x10-16), and contained a higher proportion of major histocompatibility complex (MHC) binding sites (median 5.6 binding site per kb vs 5.4; Wilcoxon-test, p-value 9.8x10-7) (Fig 2c). Further studies are required to determine if these predicted MHC binding affinity differences are associated with biologically relevant patterns of immunogenicity and T-cell responses.” (Page 10, lines 224-229)*

The authors describe an analysis of promoter usage, as informed by their RNA sequencing experiments, and conclude that alternative promoters are frequently used in gastric cancer and may lead to expression of different proteins, possibly with different functional roles. A question on the mechanistic basis of this observation immediately arises. Have the authors taken a look at methylation of the promoter regions that are alternatively used? It would be interesting to check (e.g. based on TCGA data) whether this is correlated to alternative promoter usage.

*Our response: We thank the Reviewer for this excellent suggestion. In accordance to the Reviewer’s comment, we now examined the promoters for changes in DNA methylation, using genome-wide MeDIP-seq (Methylation-dependent immunoprecipitation followed by sequencing) for 9/10 cell lines. Briefly, MeDIP reads were aligned to the human genome using bwa, and duplicates removed using samtools. DNA methylation peaks were called using MACS2 with input control. In 7/9 cell lines, we observed that isoforms when not expressed (as measured using Iso-seq data) tended to have higher methylation levels near its promoter region. In contrast, expressed isoforms tended to have reduced DNA methylation levels - this correlation was observed both in both known and novel promoters (Additional File 1; Figure S6), providing further evidence that the expressed novel promoters are bona-fide promoters as they exhibit similar epigenetic features to known expressed promoters. This analysis has now been included in the Results (Pages 18-19, lines 403-412)*

A complex part in the manuscript concerns the analysis of splicing events across known and novel isoforms using SUPPA2. For this analysis I would think that a database of reference transcripts is needed to determine in which category a splicing event belongs. How has the analysis of splice event categories be done exactly? Is this just an outcome of the SUPPA2 tool, and if so, what does the SUPPA2 tool do exactly to determine the categorization? See also Figure 3a, the figures are clear but it is unclear how these possibilities are assigned to each transcript detected in the long-read sequencing data described in this manuscript.

*Our response: In accordance with the Reviewer’s comment, we have now described the exact protocol for SUPPA2 and how SUPPA2 assigns individual splice events to specific categories, which has been included in the Methods. The amended section now reads: “After data processing, the full-length GC transcriptome was analyzed using the software SUPPA2 to detect 7 types of alternative splicing events including A3/A5 (alternative 3’ and 5’ splice sites), AF/AL (alternative first and last exons), SE (skipping exon), RI (retained intron) and MX (mutually exclusive exon). Specifically, SUPPA2 calculates possible alternative splicing events from an input annotation file containing the genomic coordinates and ranges of transcript isoforms (GTF format). We then used the generateEvent command in SUPPA2 with –f ioe options on the gtf file containing the FSM, NIC and NNC isoforms identified from SQANTI2. This command generates an ioe output file containing the local alternative splicing events from the gtf file. Splicing events were considered as ‘novel’ if all transcripts containing the splice events are novel isoforms (NIC or NNC), and splicing events found in at least one known isoform (FSM) were considered ‘known’. Expression levels of each alternative splicing event was estimated using the psiPerEvent command in SUPPA2, using the ioe file generated from generateEvent and gene expression matrix generated using Kallisto. This command generates a table containing the expression level (PSI) for each identified alternative splicing event per sample.” (Methods; Page 31-32, lines 658-673)*

*In lines 214/215 the authors describe the correlation between promoter activity predicted from short-read RNA-seq with the Iso-seq predictions. It appears that the correlation are very poor and only marginally better correlation is observed if the data are from the same cell line. I would reason that these correlations do not justify the use of short-read RNA seq data to determine promoter activity, as the authors do by use of the TCGA data for gastric cancer*

*Our response: We note this Reviewer concern. We acknowledge that while long-read methods are ideal for identification of full-length transcripts, owing to their relatively lower sequencing coverage and potential gene length biases, long-read sequencing is likely less accurate in determining transcript expression levels. This may explain the lower than expected correlation of promoter usage from the two methods, and is now cited in the Discussion as a potential limitation of our study.*

*In the revised manuscript, we have also performed a deeper analysis on this issue. Specifically, we found that when calculating correlations for isoforms with different ranges of length, we observed stronger correlations for shorter isoforms compared to longer isoforms. Importantly however, across all isoform length categories, we observed significantly higher correlations between the promoter activities inferred from Iso-seq and proActiv in the same cell line, compared to promoter activities inferred from different cell lines. This result indicates that promoter activity inferred using proActiv is most consistent with the Iso-seq data from the same cell line. We also note that the general approach used in our study, i.e. using Iso-seq full-length isoforms to generate the reference transcriptome and then quantifying isoform expression using short-read data, is commonly used in the field and thus currently represents current ‘best-practise’ [13-16]. We have now cited these additional references and amended Figure 4a to reflect the correlations based on isoforms with lengths from 0-2 Kb (Pages 13-14, lines 300-317). We hope this is acceptable.*

Minor points:

-Fig 1E: please split the bars per Number of Isoforms, so 1, 2, 3, etc, instead of grouping (2-3, 4-6)

*Our response: We have modified the Figure bars as suggested by the Reviewer.*

-Fig2A, difficult to read figure, please improve resolution/quality

*Our response: We have enhanced the quality of the figure as suggested by the Reviewer.*

-line 215, unclear sentence ("....suggesting…..novel isoforms."). Do the authors mean that promoter usage can also be identified by short-read Illumina RNA seq data, because of the (poor) correlation with Iso-seq?

*Our response: We apologize for this ambiguity and have clarified the sentence, as follows:*

*“To evaluate the accuracy of the proActiv promoter predictions, we correlated predicted promoter activities from standard RNA-seq with the predicted transcriptomes derived from Iso-seq at different ranges of isoform lengths (Additional file 1, Table S3). We observed stronger correlations for shorter isoforms compared to longer isoforms. Importantly however, at all isoform length categories, we observed significantly higher correlations between promoter activities inferred from Iso-seq and proActiv software in the same cell line, compared to promoter activities inferred from different cell lines, suggesting that promoter activity inferred using proActiv is most consistent with the Iso-seq data from the same cell line. For example, for isoforms with gene length less than 2 kb, the average correlation coefficient between the same lines was 0.63, compared to 0.49 when compared between different cell lines (Fig 4a; t-test p-value 1.8x10-12). Similar correlations were observed when restricting this analysis to either only known or novel isoforms (Additional file 1, Table S4). The moderate correlation observed between Iso-seq and proActiv from the same cell lines is likely due to the relatively lower sequencing depth and gene length biases in Iso-seq methods. The approach of using the Iso-seq full-length isoforms to generate a reference transcriptome and subsequently quantifying the isoform expression using short-read has also been used by others in the field [13-16].” (Page 13-14, lines 300-317)*

-Background: list of literature on long-read sequencing of transcripts appears incomplete, while this would be required to put their study in context. See here some examples of recent work describing long-read approaches for transcript sequencing in cancer. Please make sure the background section is complete and the recent literature is well described.

https://pubmed.ncbi.nlm.nih.gov/29954844/

https://pubmed.ncbi.nlm.nih.gov/30988345/

https://academic.oup.com/dnaresearch/article/26/1/55/5194314

*Our response: We thank the Reviewer for highlighting these relevant studies and have added these references as suggested. It is worth noting that the second reference (Lian et al., 2019) also uses a similar approach as our study to map short reads to long-read data. This is cited in the Background:*

*“Long-read sequencing has been previously used to study the full-length transcriptomes of other caner types. Analysis of SK-BR-3 breast cancer cells by genome and PacBio transcriptome sequencing led to the characterization of previously undiscovered fusion transcripts, copy-number amplifications and structural variants [17]. Another study used long-read Iso-seq and short-read RNA-seq in wild type and paclitaxel-resistant MDA-MBA-231 sublines (another breast cancer cell line) to identify novel targets of chemotherapy resistance [18]. Full-length transcriptomes can also be studied with Oxford Nanopore sequencing. For example, full-length transcripts have been sequenced from lung cancer cell lines to identify transcript variants and mutations using the MinION sequencer (Oxford Nanopore Technologies) [19].“ (Page 5, lines 108-117).*

-figure 4d: no y-axis label

*Our response: We have modified subfigure 4d as suggested by the Reviewer.*

-figure 6c: a label of the colors of the lines in the plots is lacking

*Our response: We have amended Figure 6c as suggested by the Reviewer.*

*References*

*1. Tyanova, S., T. Temu, and J. Cox, The MaxQuant computational platform for mass spectrometry-based shotgun proteomics. Nat Protoc, 2016. 11(12): p. 2301-2319.*

*2. Muratani, M., et al., Nanoscale chromatin profiling of gastric adenocarcinoma reveals cancer-associated cryptic promoters and somatically acquired regulatory elements. Nat Commun, 2014. 5: p. 4361.*

*3. Tardaguila, M., et al., SQANTI: extensive characterization of long-read transcript sequences for quality control in full-length transcriptome identification and quantification. Genome Res, 2018.*

*4. Cheneby, J., et al., ReMap 2018: an updated atlas of regulatory regions from an integrative analysis of DNA-binding ChIP-seq experiments. Nucleic Acids Res, 2018. 46(D1): p. D267-D275.*

*5. Wallenius, V., et al., Overexpression of the hepatocyte growth factor (HGF) receptor (Met) and presence of a truncated and activated intracellular HGF receptor fragment in locally aggressive/malignant human musculoskeletal tumors. Am J Pathol, 2000. 156(3): p. 821-9.*

*6. Ezzat, S., L. Zheng, and S.L. Asa, Pituitary tumor-derived fibroblast growth factor receptor 4 isoform disrupts neural cell-adhesion molecule/N-cadherin signaling to diminish cell adhesiveness: a mechanism underlying pituitary neoplasia. Mol Endocrinol, 2004. 18(10): p. 2543-52.*

*7. Shiraki, T., et al., Cap analysis gene expression for high-throughput analysis of transcriptional starting point and identification of promoter usage. Proc Natl Acad Sci U S A, 2003. 100(26): p. 15776-81.*

*8. Demircioglu, D., et al., A Pan-cancer Transcriptome Analysis Reveals Pervasive Regulation through Alternative Promoters. Cell, 2019. 178(6): p. 1465-1477 e17.*

*9. Qamra, A., et al., Epigenomic Promoter Alterations Amplify Gene Isoform and Immunogenic Diversity in Gastric Adenocarcinoma. Cancer Discov, 2017. 7(6): p. 630-651.*

*10. Cancer Genome Atlas Research, N., et al., Comprehensive Molecular Characterization of Papillary Renal-Cell Carcinoma. N Engl J Med, 2016. 374(2): p. 135-45.*

*11. Tang, S., A. Lomsadze, and M. Borodovsky, Identification of protein coding regions in RNA transcripts. Nucleic Acids Res, 2015. 43(12): p. e78.*

*12. Weirather, J.L., et al., Comprehensive comparison of Pacific Biosciences and Oxford Nanopore Technologies and their applications to transcriptome analysis. F1000Res, 2017. 6: p. 100.*

*13. Ray, T.A., et al., Comprehensive identification of mRNA isoforms reveals the diversity of neural cell-surface molecules with roles in retinal development and disease. Nat Commun, 2020. 11(1): p. 3328.*

*14. Au, K.F., et al., Characterization of the human ESC transcriptome by hybrid sequencing. Proc Natl Acad Sci U S A, 2013. 110(50): p. E4821-30.*

*15. Byrne, A., et al., Nanopore long-read RNAseq reveals widespread transcriptional variation among the surface receptors of individual B cells. Nat Commun, 2017. 8: p. 16027.*

*16. Gupta, I., et al., Single-cell isoform RNA sequencing characterizes isoforms in thousands of cerebellar cells. Nat Biotechnol, 2018.*

*17. Nattestad, M., et al., Complex rearrangements and oncogene amplifications revealed by long-read DNA and RNA sequencing of a breast cancer cell line. Genome Res, 2018. 28(8): p. 1126-1135.*

*18. Lian, B., X. Hu, and Z.M. Shao, Unveiling novel targets of paclitaxel resistance by single molecule long-read RNA sequencing in breast cancer. Sci Rep, 2019. 9(1): p. 6032.*

*19. Seki, M., et al., Evaluation and application of RNA-Seq by MinION. DNA Res, 2019. 26(1): p. 55-65.*

**Second round of review**

**Reviewer 1**

The authors have addressed all my comments. There are a few minor issues that they need to fix before publication:

1 Fig. 1g: please add y axis label.

2 Fig. 5a: please add title and x/y axis labels in this Figure panel

3 The authors added new data in the revision including proteomic and DNA methylation (MeDIP-seq) profiling. However, the data were not described in methods and data availability sections.

4. The resolution of figures presented in PDF file needs to be improved

**Reviewer 2**

The authors have considerably improved the manuscript and added novel analyses and descriptions to substantiate their findings.

I have one final comment: Axis labels appear to missing for some plots, e.g. 1G, 2B and 2C, 6F

Please carefully check the figures and make sure that all labels are correct.

**Authors Response**

**Point-by-point responses to the reviewers’ comments:**

Reviewer #1: The authors have addressed all my comments. There are a few minor issues that they need to fix before publication:

1 Fig. 1g: please add y axis label.

*We have added the y-axis for Fig. 1g*

2 Fig. 5a: please add title and x/y axis labels in this Figure panel

*We have added a title and x/y axis labels for Fig. 5a*

3 The authors added new data in the revision including proteomic and DNA methylation (MeDIP-seq) profiling. However, the data were not described in methods and data availability sections.

*We have added the methods for proteomic and MeDIP-seq profiling. We have also stated that these additional datasets are available on reasonable request from the corresponding author, similar to other statements published in Genome Biology, such as Franco et al., (2019; 20:285), Zhang et al., (2020; 21:45), and Sui et al., (2020; 21:228).*

4. The resolution of figures presented in PDF file needs to be improved

*We have now increased the resolution of all figures to 300 DPI.*

Reviewer #2:

The authors have considerably improved the manuscript and added novel analyses and descriptions to substantiate their findings.

I have one final comment: Axis labels appear to missing for some plots, e.g. 1G, 2B and 2C, 6F

*We have added axis labels for all figures.*

Please carefully check the figures and make sure that all labels are correct.

*We have checked all the figures and labels.*
